# Alcohol, drinking pattern and all-cause, cardiovascular and alcohol-related mortality in Eastern Europe

**DOI:** 10.1007/s10654-015-0092-8

**Published:** 2015-10-14

**Authors:** Martin Bobak, Sofia Malyutina, Pia Horvat, Andrzej Pajak, Abdonas Tamosiunas, Ruzena Kubinova, Galina Simonova, Roman Topor-Madry, Anne Peasey, Hynek Pikhart, Michael G. Marmot

**Affiliations:** Department of Epidemiology and Public Health, University College London, 1-19 Torrington Place, London, WC1E 6BTL UK; Institute of Internal and Preventive Medicine, Siberian Branch of the Russian Academy of Medical Sciences, Novosibirsk, Russia; Novosibirsk State Medical University, Novosibirsk, Russia; Collegium Medicum, Jagiellonian University, Kraków, Poland; Institute of Cardiology, Lithuanian University of Health Sciences, Kaunas, Lithuania; National Institute of Public Health, Prague, Czech Republic

**Keywords:** Alcohol, Mortality, Eastern Europe, Cardiovascular diseases

## Abstract

Alcohol has been implicated in the high mortality in Central and Eastern Europe but the magnitude of its effect, and whether it is due to regular high intake or episodic binge drinking remain unclear. The aim of this paper was to estimate the contribution of alcohol to mortality in four Central and Eastern European countries. We used data from the Health, Alcohol and Psychosocial factors in Eastern Europe is a prospective multi-centre cohort study in Novosibirsk (Russia), Krakow (Poland), Kaunas (Lithuania) and six Czech towns. Random population samples of 34,304 men and women aged 45–69 years in 2002–2005 were followed up for a median 7 years. Drinking volume, frequency and pattern were estimated from the graduated frequency questionnaire. Deaths were ascertained using mortality registers. In 230,246 person-years of follow-up, 2895 participants died from all causes, 1222 from cardiovascular diseases (CVD), 672 from coronary heart disease (CHD) and 489 from pre-defined alcohol-related causes (ARD). In fully-adjusted models, abstainers had 30–50 % increased mortality risk compared to light-to-moderate drinkers. Adjusted hazard ratios (HR) in men drinking on average ≥60 g of ethanol/day (3 % of men) were 1.23 (95 % CI 0.95–1.59) for all-cause, 1.38 (0.95–2.02) for CVD, 1.64 (1.02–2.64) for CHD and 2.03 (1.28–3.23) for ARD mortality. Corresponding HRs in women drinking on average ≥20 g/day (2 % of women) were 1.92 (1.25–2.93), 1.74 (0.76–3.99), 1.39 (0.34–5.76) and 3.00 (1.26–7.10). Binge drinking increased ARD mortality in men only. Mortality was associated with high average alcohol intake but not binge drinking, except for ARD in men.

**Electronic supplementary material:**

The online version of this article (doi:10.1007/s10654-015-0092-8) contains supplementary material, which is available to authorized users.

## Background

Despite decades of research, the relation between alcohol intake and mortality is not entirely clear. Most observational studies have shown a J-shaped association, with mortality lowest among light/moderate drinkers and higher risk among abstainers and heavy drinkers [1–4]. Uncertainty remains about the increased risks both in abstainers (how much of the elevated risk is due to former drinkers in this group) and among heavy drinkers (the proportion of such participants in most population studies is low and the effects of heavy drinking may be confounded by other factors associated with high alcohol intake). In addition, there is emerging evidence that in addition to volume and frequency of drinking, drinking pattern (e.g. binge drinking) may contribute to harmful effects of alcohol [5–7].

The harmful effects of alcohol and drinking patterns are an important public health issue, and it is particularly pertinent for Central and Eastern Europe (CEE) and the former Soviet Union (FSU). The estimates of alcohol intake and alcohol-related mortality in the region are high [8], alcohol intake and mortality correlate over time [9, 10], and several individual-level studies, mainly focusing on Russia, have reported increased total and cardiovascular disease (CVD) mortality in heavy drinkers [11–15].

In this report, we analysed data from a prospective cohort of a well -characterized general population sample in four countries of the region in order to investigate the association between volume, frequency and pattern of drinking with mortality from all causes, CVD, CHD and a priori defined category of alcohol-related causes of death.

## Methods

### Study populations and study subjects

The Health, Alcohol and Psychosocial factors In Eastern Europe (HAPIEE) study is a multi-centre prospective cohort study conducted in Novosibirsk (Russia), Krakow (Poland), Kaunas (Lithuania) and six middle-sized Czech towns [16]. Random population samples of 36,106 men and women aged 45–69 years at baseline in 2002–2005 (aged 49–72 in Kaunas in 2006–2008) were recruited. Response rates were 65 % in Kaunas, 61 % in Krakow and Novosibirsk and 55 % in Czech towns. After excluding participants who did not provide permission for linkage with mortality data, the analytic sample for this report consisted of 15,989 men and 18,315 women.

### Ethics, consent and permissions

The study was approved by Institutional Ethics Review Boards locally and at University College London and University College Hospital. All participants gave written informed consent.

### Follow-up

Follow-up of participants was available to 31 December 2010 in Novosibirsk and Krakow, 31 December 2011 in Czech towns and 31 December 2012 in Kaunas. Deaths were identified through record linkage with national (Czech) or local mortality registers, which recorded date of death and underlying cause of death (ICD-10 coded) [16]. In the Czech Republic, the national death register was used. In Krakow, the provincial death register was used which covers the city of Krakow and surrounding area. In Novosibirsk, the death register was established by the Institute of Internal Medicine in 1985; it uses data from the Novosibirsk office of the State Statistical Bureau (Goscomstat) and from the population registration bureau (ZAGS). In Kaunas, the regional mortality register used has previously been shown to provide a complete coverage of deaths [17]. In terms of all-cause mortality, registration of deaths is believed to be virtually complete (see WHO assessment [18]). Regarding the coding of causes of deaths, the most extensive validation has been conducted by the WHO MONICA Project [18]. As all four countries participated in MONICA (Kaunas and Novosibirsk were MONICA centres), and since the HAPIEE study adopted procedures similar to the MONICA Project, the quality of the cause of death coding, at least in broader categories such as CHD or cerebrovascular disease, is likely to be satisfactory, although specific codes (such as non-MI acute CHD or cerebral strokes) may be less reliable.

Participants who were lost to follow-up because they moved away were censored at their last date of contact. In addition to all-cause mortality, causes of death were divided into deaths from CVD (ICD-10 codes I00-99), CHD (ICD-10 codes I20-25), and pre-specified alcohol-related causes as previously defined by Zaridze et al. [14]; this group includes external causes, liver disease, liver cancer, cancer of upper aerodigestive tract, tuberculosis, pneumonia and other relevant infectious disease, non-MI acute IHD (I24), non-neoplastic pancreatic disease, and relevant ill-specified disease (see Supplementary Table 1).

### Measurements

At baseline, participants completed an extensive structured questionnaire, underwent a brief examination in a clinic, and provided a fasting blood sample. Alcohol consumption in the last 12 months was self-reported using the graduated frequency questionnaire (GFQ) [19], containing nine mutually exclusive categories of frequency (ranging from “never” to “almost every day”) and six mutually exclusive categories of amounts, in local units of beer, wine and spirits (≥10; 7–9; 5–6, 3–4; 1–2 and 0.5 drinks; one local unit equals 0.5 l of beer, 0.2 l of wine and 0.05 l of spirits or 20 g of ethanol). As only four categories of amounts (≥5; 3–4; 1–2 and 0.5 drinks) were used in Kaunas, GFQ data from all centres were harmonised using these four categories.

Several alcohol indices were derived from the harmonised GFQ for the main analyses, with the following cut-offs in men/women: (1) average daily alcohol intake (0, ≤10/5, 10–60/5–20 and ≥60/20 g/day); (2) drinking frequency (never, <1 per month, 1–3 times per month, 1–4 times per week and 5+ times per week); (3) drinking pattern, combining information on drinking frequency with quantity per occasion: light drinker (≤2/0.5 drinks per occasion), moderate drinker (≤4/2 drinks per occasion), occasional heavy drinker (>4 drinks per occasion less than once a week), and regular heavy drinker (>4 drinks per occasion more than once a week); and (4) binge drinking (≥100/60 g of ethanol in one episode at least monthly).

For all alcohol measures, participants who did not report any alcohol consumption in the past year were categorized as non-drinkers. Information on past drinking was also available in one of the centres (Novosibirsk). Additional measures on weekly alcohol intake and alcohol intake in the last 3 months from the food frequency questionnaire (FFQ) administered separately were strongly correlated with the GFQ data and therefore were not used in this report. Repeated measures of alcohol consumption, collected on average 3.5 years after the baseline examination, were available in all centres except Kaunas. Alcohol indices measured at re-examination, used in sensitivity analysis, were strongly correlated with alcohol measures at baseline, suggesting considerable stability of alcohol consumption over time. In addition, serum GGT was available for nearly all Russian participants and subsample of 4778 participants in the remaining centres, and showed expected associations with self-reported alcohol consumption in both genders.

### Covariates

Covariates, measured at baseline, included study centre, age, education (primary or less, secondary, university); current economic activity (employed/self-employed, pensioner, still working, pensioner, not working, unemployed, and other); marital status (married/cohabiting vs. single/separated/divorced/widowed); household asset index, as the number of items owned (microwave, dishwasher, washing machine, colour TV, car, freezer, satellite TV, video recorder, camcorder, mobile phone, telephone; range 0–11); subjective hardship score (summated scale derived from respondents’ reports on how often they experienced difficulties affording food, clothes and paying bills; range 0–12); smoking status (never smoked, ex-smoker, 1–10, 11–20, 21+ cigarettes a day); leisure-time physical activity (none vs. some), high depressive symptoms (yes vs. no) measured by the CESD -10;  BMI from clinical examination, and self-reported history or presence of major chronic conditions (cardiovascular disease and cancer, coded yes vs. no).

### Statistical analyses

Means with standard deviations for continuous covariates and proportions, expressed as percentages, for categorical covariates were used to describe baseline characteristics of the cohort, separately for men and women.

Cox regression was used to estimate hazard ratios (HR) with 95 % confidence intervals (CIs) for associations between alcohol measures and each mortality end-point using time-on-study as the time scale. Lowest-level drinkers were used as reference category. For binge drinking, non-binge drinkers were used as reference category. All analyses were conducted separately for men and women. We first conducted analyses separately for each centre; after checking for interactions between centres and alcohol, data from all cohorts were combined (we also report results for Novosibirsk and Kaunas vs. Krakow and Czech towns combined in Supplement because these pairs of centres are culturally similar).

For each combination of alcohol measures and mortality end-points, the first Cox model was adjusted only for age and centre, and the second model was additionally adjusted for all remaining covariates. We also repeated the analyses excluding participants with major chronic conditions at baseline but the results were similar. For each Cox model, proportional hazards assumption was checked by including interactions with log(time) and by examination of Schoenfeld residuals. A suggestion of violation of proportional hazards assumption with age was dealt with by including its interaction with time but this had no appreciable effect on associations between alcohol and mortality.

Missing data on baseline covariates were filled in using multiple imputations by chained equations implemented in Stata 13. Imputation models included vital status, alcohol, covariates and a small number of auxiliary variables, predictive either of missingness or values of incomplete variables, and the Nelson-Aalen estimator of cumulative baseline hazard [20]. Ten imputed datasets were generated and Rubin’s combination rules were used to obtain Cox regression estimates from the multiply imputed data. All statistical analyses were conducted in Stata 13.

## Results

Over the median follow up of 6.9 years (or 230,246 person-years), 2895 deaths from all causes occurred among 34,304 participants included in the analysis; about 43.0 % of all deaths were from CVD (n = 1222), 672 deaths were from CHD and 489 from pre-specified alcohol-related conditions (Table 1). At baseline, 12 % of men reported to be non-drinkers, 3 % reported to drink on average ≥60 g of ethanol per day and 21 % were classified as binge drinkers; among women, 23 % were non-drinkers, 2 % drank ≥20 g of ethanol per day and 7 % were binge drinkers. Deaths and descriptive characteristics of study participants by centre are shown in Supplementary Tables 2 and 3, respectively. Associations of the GFQ-based alcohol indices with other alcohol measures, serum GGT and with repeated assessment are shown in Supplementary Table 4.

**Table 1:** Participant numbers and deaths by cause and sex

	Participant numbers		Mortality end-points							
	Total		All-cause		CVD (I00-99)		CHD (I20-5)		Pre-specified alcohol-related^b^	
	n	%	n	%	n	%	n	%	n	%
*Men (n* *=* *15,960)*										
Alcohol intake										
Non-drinker	1991	12.6	364	19.1	160	19.4	99	20.2	63	17.4
<10 g/day	8685	55.0	986	51.7	448	54.3	258	52.5	158	43.5
10–60 g/day	4686	29.6	495	25.9	187	22.7	115	23.4	120	33.1
≥60 g/day	443	2.8	64	3.4	30	3.6	19	3.9	22	6.1
n	15,805	100	1909	100	825	100	491	100	363	100
Drinking frequency										
Never	1991	12.6	364	19.1	160	19.4	99	20.2	63	17.4
<1 month	2231	14.1	284	14.9	134	16.2	81	16.5	33	9.1
1–3 month	3901	24.7	450	23.6	210	25.5	123	25.1	73	20.1
1–4 week	5269	33.3	546	28.6	215	26.1	126	25.7	126	34.7
5+ week	2413	15.3	265	13.9	106	12.8	62	12.6	68	18.7
n	15,805	100	1909	100	825	100	491	100	363	100
Drinking pattern^a^										
Non-drinker	1991	12.6	364	19.1	160	19.4	99	20.2	63	17.4
Light (≤2 drinks/occasion)	3751	23.7	440	23.0	184	22.3	102	20.8	72	19.8
Moderate (≤4 drinks/occasion)	3348	21.2	407	21.3	183	22.2	108	22.0	69	19.0
Occasional heavy (>4 drinks/occasion <1 week)	5558	35.2	556	29.1	237	28.7	144	29.3	117	32.2
Regular heavy (>4 drinks/occasion ≥1 week)	1157	7.3	142	7.4	61	7.4	38	7.7	42	11.6
n	15,805	100	1909	100	825	100	491	100	363	100
Binge drinking (100 g ≥1 month)										
Non-drinker	1991	12.6	364	19.1	160	19.4	99	20.2	63	17.4
Non-binge drinker	10,436	66.0	1162	60.9	503	61.0	293	59.7	200	55.1
Binge drinker	3378	21.4	383	20.1	162	19.6	99	20.2	100	27.5
n	15,805	100	1909	100	825	100	491	100.1	363	100
*Women (n* *=* *18,299)*										
Alcohol intake										
Non-drinker	4245	23.5	352	37.9	162	42.9	69	39.7	34	28.6
<5 g/day	11,779	65.2	500	53.9	188	49.7	96	55.2	69	58.0
5–20 g/day	1687	9.3	53	5.7	22	5.8	7	4.0	10	8.4
≥20 g/day	343	1.9	23	2.5	6	1.6	2	1.1	6	5.0
n	18,054	100	928	100	378	100	174	100	119	100
Drinking frequency										
Never	4245	23.5	352	37.9	162	42.9	69	39.7	34	28.6
<1 month	5977	33.1	289	31.1	126	33.3	62	35.6	37	31.1
1–3 month	5087	28.2	189	20.4	54	14.3	31	17.8	29	24.4
1–4 week	2273	12.6	73	7.9	27	7.1	10	5.7	15	12.6
5+ week	472	2.6	25	2.7	9	2.4	2	1.1	4	3.4
n	18,054	100	928	100	378	100	174	100	119	100
Drinking pattern^a^										
Non-drinker	4245	23.5	352	37.9	162	42.9	69	39.7	34	28.6
Light (≤0.5 drink/occasion)	2656	14.7	137	14.8	53	14.0	25	14.4	13	10.9
Moderate (≤2 drinks/occasion)	7152	39.6	293	31.6	117	31.0	62	35.6	41	34.5
Occasional heavy (>2 drinks/occasion <1 week)	3768	20.9	133	14.3	45	11.9	18	10.3	25	21.0
Regular heavy (>2 drinks/occasion ≥1 week)	233	1.3	13	1.4	1	0.3	0	0.0	6	5.0
n	18,054	100	928	100	378	100.1	174	100	119	100
Binge drinking (60 g ≥1 month)										
Non-drinker	4245	23.5	352	37.9	162	42.9	69	39.7	34	28.6
Non-binge drinker	12,592	69.7	531	57.2	198	52.4	98	56.3	74	62.2
Binge drinker	1217	6.7	45	4.8	18	4.8	7	4.0	11	9.2
n	18,054	100	928	99.9	378	100.1	174	100	119	100

^a^Standard drink = 20 g of pure alcohol

^b^Pre-specified alcohol-related causes of death include external causes, liver disease, including cancer, cancer of upper aerodigestive tract, tuberculosis, pneumonia and other relevant infectious disease, non-MI acute IHD (I24), non-neoplastic pancreatic disease, and relevant ill-specified disease as in Zaridze et al. [14]

The associations between alcohol consumption indices and mortality risk are shown in Tables 2 (men) and 3 (women). Among men, non-drinkers and those drinking ≥60 g of ethanol per day had the highest mortality. For all-cause mortality, the adjusted HRs were 1.36 for non-drinkers and 1.23 in the highest intake group. The adjusted HRs were highest for the pre-specified alcohol related causes but they were also elevated for CVD and CHD. Daily alcohol intake was not associated with non-CHD cardiovascular deaths in men but in female non-drinkers the adjusted HRs were highest for this group of deaths (Supplementary Table 5). There were too few deaths in specific non-CHD cardiovascular categories (e.g. cerebral stroke) to allow for a further examination of associations with alcohol (see Supplementary Table 5). Frequent drinking and drinking pattern were not associated with mortality except pre-specified alcohol-related deaths. In women, mortality risks were also elevated in non-drinkers and the heaviest intake category but the HRs were higher than among men; female regular heavy drinkers had considerably increased risk of death from alcohol-related causes but the number of deaths in this group was small. In both sexes, binge drinking was weakly associated with mortality from ARD in men but not with all-cause or CVD mortality.

**Table 2:** Cox regression results for alcohol consumption and mortality end-points in men (n = 15,989)

	All-cause mortality				CVD (I00-99) mortality				CHD (I20-5) mortality				Alcohol-related mortality^b^			
	Age adjusted^c^		Fully adjusted^d^		Age adjusted		Fully adjusted		Age adjusted		Fully adjusted		Age adjusted		Fully adjusted	
	HR	95 % CI	HR	95 % CI	HR	95 % CI	HR	95 % CI	HR	95 % CI	HR	95 % CI	HR	95 % CI	HR	95 % CI
Alcohol intake																
Non-drinker	1.63	[1.44–1.84]	1.36	[1.20–1.54]	1.60	[1.33–1.92]	1.32	[1.09–1.58]	1.70	[1.34–2.16]	1.42	[1.12–1.80]	1.70	[1.26–2.28]	1.39	[1.03–1.87]
<10 g/day	1.00		1.00		1.00		1.00		1.00		1.00		1.00		1.00	
10–60 g/day	1.03	[0.92–1.15]	1.00	[0.90–1.12]	0.85	[0.71–1.01]	0.86	[0.73–1.03]	0.91	[0.73–1.14]	0.92	[0.74–1.16]	1.40	[1.10–1.78]	1.34	[1.05–1.71]
≥60 g/day	1.60	[1.24–2.06]	1.23	[0.95–1.59]	1.71	[1.18–2.49]	1.38	[0.95–2.02]	2.01	[1.26–3.23]	1.64	[1.02–2.64]	2.93	[1.86–4.62]	2.03	[1.28–3.23]
Drinking frequency																
Never	1.57	[1.34–1.84]	1.34	[1.14–1.57]	1.52	[1.20–1.92]	1.29	[1.02–1.63]	1.54	[1.14–2.07]	1.31	[0.97–1.77]	2.11	[1.38–3.22]	1.79	[1.17–2.73]
<1 month	1.00		1.00		1.00		1.00		1.00	.	1.00		1.00		1.00	
1–3 month	0.99	[0.85–1.15]	0.99	[0.85–1.15]	0.98	[0.79–1.22]	0.99	[0.80–1.24]	0.93	[0.71–1.24]	0.94	[0.71–1.25]	1.26	[0.84–1.89]	1.28	[0.85–1.93]
1–4 week	0.95	[0.82–1.10]	0.98	[0.84–1.13]	0.81	[0.65–1.00]	0.86	[0.69–1.07]	0.77	[0.58–1.02]	0.81	[0.61–1.08]	1.60	[1.09–2.35]	1.68	[1.15–2.47]
5+ week	1.06	[0.89–1.25]	1.02	[0.86–1.21]	0.98	[0.76–1.28]	1.01	[0.77–1.31]	1.03	[0.74–1.45]	1.06	[0.75–1.49]	2.05	[1.35–3.12]	1.94	[1.27–2.96]
Drinking pattern^a^																
Non-drinker	1.65	[1.44–1.91]	1.38	[1.19–1.59]	1.68	[1.36–2.09]	1.40	[1.13–1.74]	1.79	[1.35–2.37]	1.50	[1.13–1.99]	1.62	[1.15–2.28]	1.31	[0.93–1.85]
Light (QPO ≤40 g)	1.00		1.00		1.00		1.00		1.00		1.00		1.00		1.00	
Moderate (QPO ≤80 g)	1.04	[0.91–1.20]	1.05	[0.92–1.21]	1.04	[0.84–1.28]	1.09	[0.88–1.34]	1.06	[0.80–1.39]	1.12	[0.85–1.47]	1.02	[0.73–1.42]	1.02	[0.73–1.43]
Occasional heavy (QPO >80 g <1 pw)	1.03	[0.90–1.17]	1.00	[0.88–1.14]	1.02	[0.84–1.25]	1.04	[0.85–1.27]	1.06	[0.82–1.38]	1.07	[0.82–1.39]	1.12	[0.83–1.52]	1.08	[0.80–1.47]
Regular heavy (QPO >80 g ≥1 pw)	1.19	[0.98–1.45]	1.02	[0.84–1.25]	1.10	[0.82–1.49]	1.03	[0.76–1.39]	1.16	[0.79–1.70]	1.08	[0.73–1.59]	1.72	[1.16–2.56]	1.37	[0.92–2.04]
Binge drinking (100 g ≥1 month)																
Non-drinker	1.65	[1.46–1.86]	1.36	[1.20–1.54]	1.68	[1.40–2.01]	1.35	[1.13–1.63]	1.74	[1.38–2.20]	1.42	[1.12–1.79]	1.62	[1.21–2.16]	1.30	[0.97–1.74]
Non-binge drinker	1.00		1.00		1.00		1.00		1.00		1.00		1.00		1.00	
Binge drinker	1.16	[1.03–1.30]	1.02	[0.91–1.16]	1.09	[0.91–1.30]	1.00	[0.84–1.21]	1.08	[0.86–1.37]	0.99	[0.78–1.25]	1.51	[1.18–1.93]	1.27	[0.99–1.63]
N	15,989		15,989		15,989		15,989		15,989		15,989		15,989		15,989	
No. of cases	1939		1939		833		833		493		493		369		369	
Person years (000s)	105.02		105.02		105.02		105.02		105.02		105.02		105.02		105.02	

^a^QPO = Quantity per occasion

^b^Pre-specified alcohol-related causes of death include external causes, liver disease, including cancer, cancer of upper aerodigestive tract, tuberculosis, pneumonia and other relevant infectious disease, non-MI acute IHD (I24), non-neoplastic pancreatic disease, and relevant ill-specified disease as in Zaridze et al. [14]

^c^All models are adjusted for study center

^d^Fully-adjusted model includes age, education, marital status, economic activity, asset score, subjective hardship score, smoking, physical activity, BMI, prevalent CVD and cancer, and depressive symptoms

**Table 3:** Cox regression results for alcohol consumption and mortality end-points in women (n = 18,315)

	All-cause mortality				CVD (I00-99) mortality				CHD (I20-5) mortality				Alcohol-related mortality^b^			
	Age adjusted^c^		Fully adjusted^d^		Age adjusted		Fully adjusted		Age adjusted		Fully adjusted		Age adjusted		Fully adjusted	
	HR	95 % CI	HR	95 % CI	HR	95 % CI	HR	95 % CI	HR	95 % CI	HR	95 % CI	HR	95 % CI	HR	95 % CI
Alcohol intake																
Non-drinker	1.64	[1.41–1.90]	1.35	[1.16–1.57]	2.00	[1.59–2.50]	1.57	[1.25–1.97]	1.87	[1.34–2.60]	1.40	[1.01–1.96]	1.30	[0.84–2.02]	1.00	[0.64–1.56]
<5 g/day	1.00		1.00		1.00		1.00		1.00		1.00		1.00		1.00	
5–20 g/day	0.89	[0.66–1.19]	0.91	[0.67–1.22]	1.21	[0.77–1.90]	1.22	[0.77–1.93]	0.88	[0.41–1.91]	0.92	[0.42–1.99]	1.01	[0.51–1.97]	1.01	[0.51–1.99]
≥20 g/day	2.04	[1.34–3.12]	1.92	[1.25–2.93]	1.86	[0.82–4.24]	1.74	[0.76–3.99]	1.42	[0.35–5.81]	1.39	[0.34–5.76]	3.00	[1.28–7.04]	3.00	[1.26–7.10]
Drinking frequency																
Never	1.58	[1.34–1.86]	1.35	[1.15–1.59]	1.78	[1.39–2.28]	1.46	[1.14–1.87]	1.78	[1.24–2.55]	1.42	[0.98–2.04]	1.27	[0.78–2.08]	1.04	[0.63–1.70]
<1 month	1.00		1.00		1.00		1.00		1.00		1.00		1.00		1.00	
1–3 month	0.93	[0.77–1.12]	1.00	[0.83–1.21]	0.71	[0.52–0.98]	0.78	[0.56–1.07]	0.85	[0.55–1.32]	0.98	[0.63–1.51]	0.98	[0.60–1.61]	1.07	[0.65–1.75]
1–4 week	0.87	[0.67–1.13]	0.98	[0.75–1.27]	0.96	[0.63–1.47]	1.12	[0.73–1.71]	0.88	[0.45–1.72]	1.08	[0.55–2.13]	1.09	[0.59–2.01]	1.24	[0.66–2.31]
5+ week	1.31	[0.87–1.98]	1.36	[0.90–2.06]	1.43	[0.72–2.84]	1.43	[0.72–2.87]	0.84	[0.20–3.46]	0.92	[0.22–3.85]	1.28	[0.45–3.68]	1.52	[0.53–4.41]
Drinking pattern^a^																
Non-drinker	1.53	[1.25–1.87]	1.29	[1.06–1.58]	1.80	[1.31–2.47]	1.47	[1.07–2.02]	1.72	[1.08–2.72]	1.36	[0.86–2.16]	1.64	[0.86–3.12]	1.30	[0.68–2.48]
Light (QPO ≤10 g)	1.00		1.00		1.00		1.00		1.00		1.00		1.00		1.00	
Moderate (QPO ≤40 g)	0.87	[0.71–1.07]	0.93	[0.75–1.14]	0.88	[0.63–1.22]	0.95	[0.68–1.32]	0.90	[0.57–1.44]	0.99	[0.62–1.58]	1.25	[0.66–2.36]	1.34	[0.71–2.52]
Occasional heavy (QPO >40 g <1 pw)	0.98	[0.77–1.25]	0.96	[0.75–1.23]	1.00	[0.66–1.50]	0.95	[0.63–1.44]	0.84	[0.46–1.56]	0.83	[0.45–1.54]	1.54	[0.77–3.07]	1.50	[0.75–3.02]
Regular heavy (QPO >40 g ≥1 pw)	1.70	[0.96–3.01]	1.39	[0.79–2.47]	0.49	[0.07–3.53]	0.38	[0.05–2.74]	NA		NA		5.82	[2.17–15.61]	4.87	[1.79–13.24]
Binge drinking (60 g ≥1 month)																
Non-drinker	1.66	[1.44–1.92]	1.36	[1.18–1.58]	2.01	[1.61–2.52]	1.58	[1.26–1.98]	1.92	[1.38–2.68]	1.44	[1.03–2.01]	1.30	[0.84–2.01]	0.99	[0.64–1.54]
Non-binge drinker	1.00		1.00		1.00		1.00		1.00		1.00		1.00		1.00	
Binge drinker	1.25	[0.92–1.70]	1.11	[0.82–1.52]	1.64	[1.01–2.68]	1.41	[0.86–2.31]	1.42	[0.65–3.09]	1.27	[0.58–2.80]	1.65	[0.87–3.15]	1.40	[0.73–2.70]
N	18,315		18,315		18,315		18,315		18,315		18,315		18,315		18,315	
No. of cases	956		956		389		389		179		179		120		120	
Person years (000s)	125.04		125.04		125.04		125.04		125.04		125.04		125.04		125.04	

^a^QPO = Quantity per occasion

^b^Pre-specified alcohol-related causes of death include external causes, liver disease, including cancer, cancer of upper aerodigestive tract, tuberculosis, pneumonia and other relevant infectious disease, non-MI acute IHD (I24), non-neoplastic pancreatic disease, and relevant ill-specified disease as in Zaridze et al. [14]

^c^All models are adjusted for study center

^d^Fully-adjusted model includes age, education, marital status, economic activity, asset score, subjective hardship score, smoking, physical activity, BMI, prevalent CVD and cancer, and depressive symptoms

We have conducted a number of sensitivity analyses, reported in supplementary material. Restricting the analytical sample to participants without existing chronic diseases at baseline produced effects similar to adjusted models (Supplementary Tables 6 and 7). Results for the Czech and Polish cohorts vs. the Russian and Lithuanian cohorts are shown in Supplementary Tables 8 and 9; the differences between these two sets of populations were not statistically significant. In the Russian cohort, where information on former drinking was available, the excess risk in non-drinkers was larger in female (but not male) former drinkers (Supplementary Table 10).

Using the prevalence of heavy drinking and the associated adjusted hazard ratios, the population attributable risk fractions (PARF) for all-cause mortality were 2.8 % in men and 5.5 % in women; PARF calculated under different assumptions for all cohorts combined and for the Russian cohort are shown in Supplementary Table 11. Finally, we estimated how much of the hazard ratios for mortality in the Russian cohort compared to the Czech cohort can be explained by alcohol. In men, the age-adjusted HR was 1.92 [1.69–2.18] and 1.88 [1.65–2.14] after additionally controlling for alcohol consumption; in women, these two HRs were 1.21 [1.01–1.45] and 1.23 [1.02–1.48].

## Discussion

This prospective study in well characterized middle-aged and older Central and Eastern European participants found associations between high alcohol intake and increased mortality from all causes and CVD in both men and women. Binge drinking was significantly associated with ARD in women but not with CVD and total mortality in either gender. Due to the low proportions of heavy drinkers in these cohorts, population risk fractions of mortality attributable to alcohol were modest, under 6 %. Alcohol did not seem to explain differences in all-cause mortality between cohorts.

Several limitations of this study should be considered when interpreting the results. First, these urban population samples are not necessarily representative of whole countries. That said, their mortality rates and trends and risk factors are similar to national data [21–24] and two of the four study centres participated in the MONICA study which remains the main source of validated international data on CVD [25]. Second, the response rates were moderate (although similar to most contemporary population-based cohorts) and this may affect the representativeness. In particular, responders were healthier and had higher education than non-responders, and this may have led to underestimation of the prevalence of heavy drinkers in the cohort participants. On the other hand, the prevalence of heavy drinking in persons aged 55–74 years in a recently published Russian cohort with higher response rate [14] was similar to our study. Moreover, it is unlikely that the selection bias would affect the internal consistency of the findings regarding the association between exposure and outcome, although it could affect estimates of the population attributable risk fraction (as discussed below).

Third, measurement of alcohol consumption is notoriously difficult; this study, similarly to most other studies, relied on participants’ self-report. Although some underreporting is likely, particularly among women [26, 27], our main alcohol variables, based on GFQ, were strongly associated with other measures, including weekly intake, food frequency questionnaire (administered separately), serum GGT concentrations and repeated assessment of alcohol intake. The validity of the alcohol measurements is further supported by their strong association with pre-specified alcohol-related deaths, the strength of which was similar to that reported previously [14].

Fourth, as the participants in these cohorts were 45 years old and older and, as drinking in most populations declines with age, there were fewer heavy drinkers than in the younger age groups. The prevalence of heavy drinking, however, was similar to that reported by the Zaridze et al. [14], as well as to previous Novosibirsk population samples [11, 28, 29].

As well as limitations, our study also has important strengths. First, the study participants were well characterized, with detailed assessment of alcohol intake and potential confounders, repeated assessment of alcohol consumption and with one (albeit imperfect) biomarker associated with alcohol. This provides confidence that, even if absolute alcohol intakes were underestimated, the rank of participants by intake is reasonably reliable. Second, this study included four population samples with different mortality rates and different drinking patterns. The consistency of the results across cohorts supports the reliability of the findings. Third, since we had access to death registers covering the whole population, the follow up is virtually complete. Due to differential access to register data, the length of follow up differs between cohorts but this should not affect the results.

Our findings are consistent with the evidence that both non-drinkers and heavy drinkers have increased risk of all-cause and cardiovascular mortality [1–4]. Regarding the evidence on alcohol and mortality in Eastern Europe and Russia, the effects observed in this cohort are similar to a recently reported large Siberian cohort [14] and to an earlier Novosibirsk cohort [11]. Although our cut-off for “heavy drinking” was by some 7 g per day lower than in the aforementioned Siberian cohort [14], the relative risks were of a similar magnitude, both for all-causes and for alcohol-related causes of death. Several smaller cohort studies reported either smaller or larger effects [15, 30–32]; these inconsistencies can be due to low power and methodological differences. [33].

Increased mortality risk in non-drinkers relative to light/moderate drinkers observed in our study is consistent with the J-shaped dose–response association between daily alcohol consumption and mortality which has been widely reported by observational studies [33]. This could partly reflect inclusion of former drinkers among non-drinkers. Baseline reports on past drinking from the Novosibirsk HAPIEE centre (these data were not available in other centres) showed that among non-drinkers (14 and 18 % of men and women, respectively), 92 % of men and 53 % of women reported drinking in the past, with over 20 % quitting drinking due to health-related reasons, including cardiovascular disease. However, in Novosibirsk male ex-drinkers did not have significantly increased mortality in fully-adjusted models compared to stable drinkers but female ex-drinkers did (Supplementary Table 10). Because we could not distinguish current non-drinkers who are ex-drinkers from lifelong abstainers in all study centres, we used light drinkers as the reference category.

It is not clear how the findings from cohort studies relate to higher estimates of both absolute and relative effects of alcohol in Russia reported in case–control studies, [12] particularly in a study focusing on hazardous drinking defined as consumption of surrogate alcohol (beverages not primarily intended for human consumption, such as antifreeze or aftershaves) [13]. We did not assess such hazardous drinking at baseline but at re-examination it was reported by only 7 subjects. Our study is thus not well placed to study the effects of surrogates. Drinkers of such substances are unlikely to participate in cohort studies and these harmful effects would be missed. On the other hand, such drinkers are likely to suffer from multiple disadvantages (social isolation and exclusion, unemployment, material deprivation, depression) which can contribute to their increased mortality risk.

The absence of an effect of drinking pattern is noteworthy. There is a growing literature suggesting that, in addition to regular heavy drinking, episodic heavy drinking is associated with increased total and CVD mortality [5, 6, 34]. We tried a number of ways to specify drinking patterns in our data. Except for alcohol-related causes of death, we did not detect significant effects of binge drinking on mortality, although the graduate frequency questionnaire is better suited to assess drinking pattern than the traditional separate questions on average or last week intake and the frequency of any drinking [19]. The absence of an effect on mortality is consistent with the finding that binge drinking in these cohorts was not associated with blood pressure (while there was a strong effect of drinking volume) [35], and with an earlier Novosibirsk cohort which also failed to find strong effect of drinking pattern [11].

Most previous studies on alcohol in Eastern Europe focused on males. Our findings and the studies by Zaridze et al. [13, 14] suggest that the effects of heavy drinking in women are of a similar magnitude as in men. Interestingly, the Arkhangelsk study reported increased mortality only in female drinkers, not in males [30].

It has been suggested that some CVD deaths in Russia could be wrongly coded alcohol poisoning [36]. For this reason, Zaridze et al. included acute non-MI CHD deaths (ICD-10 code I24) into the alcohol-related causes [14]. There were only 7 such deaths in our study; instead, we were more concerned about potential miscoding of deaths from chronic CHD (ICD-10 code I25) in Novosibirsk. However, the association of these deaths with alcohol was similar to that of all CVD deaths and weaker than with alcohol-related causes, not supporting the hypothesis that a particular category of CVD deaths were systematically misdiagnosed in relation to alcohol intake.

An important issue is the population impact of alcohol on mortality. Alcohol has been widely seen as a major cause of premature deaths in Russia, particularly at younger ages [13, 14, 37, 38]. Our data, and our approximate estimations based on results by Zaridze et al. [14], suggest population attributable risk fractions around or under 10 % for all-cause mortality in both the previously reported Russian cohort in the age group 55–74 years [14] and in this study. These figures are probably underestimates, because of measurement error (leading to underestimation of relative risk), underestimation of alcohol intake (particularly in women [26, 27]), and the fact that participants aged 45 years and older were drinking less than at their lifetime peak alcohol intake. Nevertheless, our estimates appear substantially lower than previously reported [12, 13, 15]. This is consistent with the finding was that although– in line with national statistics– the male mortality in Russia was double of that in the Czech and Polish cohorts, alcohol did not explain a major part of this excess. As the response rates and other issues potentially affecting attributable mortality estimation are similar across cohorts, the unexplained high male mortality in the Russian cohort, compared to the other three cohorts, is unlikely to be due to these biases.

## Conclusions

There is no doubt that alcohol is an important cause of mortality in Eastern Europe and globally, and the associations of alcohol with mortality in this study were broadly in line with those reported elsewhere. It remains uncertain, however, whether the high long-term mortality rates of middle aged and older persons in Russia are caused predominantly by alcohol and what is the contribution of other factors.

## Electronic supplementary material

Supplementary material 1 (docx 206 kb)
