# Supplementary material for: Alcohol, drinking pattern and all-cause, cardiovascular and alcohol-related mortality in Eastern Europe
Source: Eur J Epidemiol. 2015 Oct 14;31:21–30. doi: 10.1007/s10654-015-0092-8 (PMC4756032; doi:10.1007/s10654-015-0092-8)
Supplement: Supplementary file 1 — Supplementary material 1 (docx 206 kb) [file 10654_2015_92_MOESM1_ESM.docx]

**Supplementary appendix** accompanying manuscript:

**“Alcohol, drinking pattern and all-cause, cardiovascular and alcohol-related mortality in Eastern Europe”**

Contents

[Appendix Table 1. Number of deaths by category and ICD-10 code(s) 2](#_Toc429407706)

[Appendix Table 2. Participant numbers, person years of follow-up and deaths by cause, sex and center 3](#_Toc429407707)

[Appendix Table 3. Baseline descriptive characteristics of participants by sex and center 5](#_Toc429407708)

[Appendix Table 4. Associations of baseline graduated frequency-based alcohol indices with other and repeated alcohol measures and serum GGT levels 6](#_Toc429407709)

[Appendix Table 5. Cox regression results for alcohol consumption and deaths from non-CHD cardiovascular causes 8](#_Toc429407710)

[Appendix Table 6. Cox regression results for alcohol consumption and mortality end-points in men free of CVD and cancer at baseline (n=12,357) 9](#_Toc429407711)

[Appendix Table 7. Cox regression results for alcohol consumption and mortality end-points in women free of CVD and cancer at baseline (n=14,233) 10](#_Toc429407712)

[Appendix Table 8. Cox regression results for alcohol consumption and mortality end-points in Novosibirsk & Kaunas vs. Czech & Krakow men 11](#_Toc429407713)

[Appendix Table 9. Cox regression results for alcohol consumption and mortality end-points in Novosibirsk & Kaunas vs. Czech & Krakow women 12](#_Toc429407714)

[Appendix Table 10. Cox regression results for past drinking behavior (before baseline) and mortality end-points in Novosibirsk 13](#_Toc429407715)

[Population attributable risk fraction (PARF) calculations 14](#_Toc429407716)

[Appendix Table 11. Population attributable risk fraction (PARF) calculations for alcohol and all-cause mortality under actual and hypothetical scenarios in men in all centers and Novosibirsk 14](#_Toc429407717)

# Appendix Table 1. Number of deaths by category and ICD-10 code(s)

|  | **Number of deaths** | |
| --- | --- | --- |
|  | **Men (n=15,989)** | **Women (n=18,315)** |
| **Cause of death ICD-10 code(s)** |  |  |
| **Alcohol-related causes (ARD)** |  |  |
| External causes F10, S00-T99, V00-Y99 | 130 | 32 |
| Alcohol poisoning F10, T51, X45, Y15 | 19 | 2 |
| Suicide X60-84 | 24 | 8 |
| Assault X85-Y09, Y35-36 | 5 | 1 |
| Transport accidents V00-99 | 10 | 5 |
| Other external (not included above) | 72 | 16 |
| Liver cancer C22 | 19 | 7 |
| Other liver disease B15-19, K70-77, I85 | 61 | 35 |
| Upper aerodigestive cancer C00-15, 32 | 41 | 5 |
| Tuberculosis A15-19, B90 | 4 | 1 |
| Pneumonia, etcb J00-39, J60-98 | 35 | 18 |
| Non-MI acute IHD I24 | 5 | 2 |
| Non-neoplastic pancreatic disease K85-86 | 14 | 7 |
| Ill-specified disease R00-99 | 56 | 17 |
| **Other vascular diseases** |  |  |
| Acute MI or angina I20-23 | 125 | 40 |
| Chronic IHD I25 | 376 | 143 |
| Stroke I60-69 | 166 | 106 |
| Other vascular disease I00-99 (not included above) | 161 | 98 |
| **Other neoplastic diseases** |  |  |
| Lung cancer C33-34 | 187 | 50 |
| Breast cancer C50 | 0 | 58 |
| Colorectal cancer C18-21, 26 | 79 | 56 |
| Pancreatic cancer C25 | 30 | 26 |
| Stomach cancer C16 | 54 | 25 |
| Cancer site unspecified C76-80 | 33 | 13 |
| Other neoplastic diseases (not included above) | 236 | 149 |
| **Other diseases** |  |  |
| Gastro-oesophageal diseaseb K20-31, 92 | 17 | 3 |
| Peritonitis K65 | 3 | 0 |
| COPDb I27, J40-47 | 38 | 11 |
| Any remaining causes (not included above) | 69 | 54 |
| **All causes A00-T98, V01-Z99** | **1939** | **956** |

# Appendix Table 2. Participant numbers, person years of follow-up and deaths by cause, sex and center

|  | **Czech towns** | | | | |  |  | **Novosibirsk** | | | | |  |  | **Krakow** | | | | |  |  | **Kaunas** | | | | |
| --- | --- | --- | --- | --- | --- | --- | --- | --- | --- | --- | --- | --- | --- | --- | --- | --- | --- | --- | --- | --- | --- | --- | --- | --- | --- | --- |
|  | **Mortality end-points** | | | | | | | | | | | | | | | | | | | | | | | | | |
|  | **N** | **All-**  **cause** | **CVD** | **CHD** | **ARD** |  |  | **N** | **All-**  **cause** | **CVD** | **CHD** | **ARD** |  |  | **N** | **All-**  **cause** | **CVD** | **CHD** | **ARD** |  |  | **N** | **All-**  **cause** | **CVD** | **CHD** | **ARD** |
| **Men** |  |  |  |  |  |  |  |  |  |  |  |  |  |  |  |  |  |  |  |  |  |  |  |  |  |  |
| Alcohol volume |  |  |  |  |  |  |  |  |  |  |  |  |  |  |  |  |  |  |  |  |  |  |  |  |  |  |
| Non-drinker | 228 | 64 | 32 | 17 | 10 |  |  | 557 | 109 | 59 | 40 | 19 |  |  | 1036 | 163 | 58 | 34 | 27 |  |  | 170 | 28 | 11 | 8 | 7 |
| <10 g/d | 1817 | 199 | 80 | 37 | 31 |  |  | 2035 | 324 | 194 | 120 | 47 |  |  | 2811 | 255 | 91 | 51 | 52 |  |  | 2022 | 208 | 83 | 50 | 28 |
| 10-60 g/d | 1422 | 141 | 43 | 18 | 28 |  |  | 1439 | 189 | 82 | 59 | 55 |  |  | 881 | 87 | 31 | 18 | 22 |  |  | 944 | 78 | 31 | 20 | 15 |
| ≥60 g/d | 206 | 24 | 11 | 5 | 6 |  |  | 96 | 24 | 13 | 8 | 10 |  |  | 75 | 10 | 5 | 5 | 3 |  |  | 66 | 6 | 1 | 1 | 3 |
| Observations | 3673 | 428 | 166 | 77 | 75 |  |  | 4127 | 646 | 348 | 227 | 131 |  |  | 4803 | 515 | 185 | 108 | 104 |  |  | 3202 | 320 | 126 | 79 | 53 |
|  |  |  |  |  |  |  |  |  |  |  |  |  |  |  |  |  |  |  |  |  |  |  |  |  |  |  |
| Drinking frequency |  |  |  |  |  |  |  |  |  |  |  |  |  |  |  |  |  |  |  |  |  |  |  |  |  |  |
| Never | 228 | 64 | 32 | 17 | 10 |  |  | 557 | 109 | 59 | 40 | 19 |  |  | 1036 | 163 | 58 | 34 | 27 |  |  | 170 | 28 | 11 | 8 | 7 |
| <1 month | 518 | 56 | 13 | 4 | 7 |  |  | 566 | 94 | 54 | 34 | 13 |  |  | 692 | 76 | 39 | 22 | 8 |  |  | 455 | 58 | 28 | 21 | 5 |
| 1-3 month | 625 | 83 | 38 | 21 | 14 |  |  | 1063 | 177 | 105 | 64 | 26 |  |  | 1137 | 91 | 30 | 18 | 22 |  |  | 1076 | 99 | 37 | 20 | 11 |
| 1-4 week | 1135 | 109 | 40 | 17 | 23 |  |  | 1590 | 212 | 104 | 71 | 51 |  |  | 1392 | 123 | 36 | 16 | 28 |  |  | 1152 | 102 | 35 | 22 | 24 |
| 5+ week | 1167 | 116 | 43 | 18 | 21 |  |  | 351 | 54 | 26 | 18 | 22 |  |  | 546 | 62 | 22 | 18 | 19 |  |  | 349 | 33 | 15 | 8 | 6 |
| Observations | 3673 | 428 | 166 | 77 | 75 |  |  | 4127 | 646 | 348 | 227 | 131 |  |  | 4803 | 515 | 185 | 108 | 104 |  |  | 3202 | 320 | 126 | 79 | 53 |
|  |  |  |  |  |  |  |  |  |  |  |  |  |  |  |  |  |  |  |  |  |  |  |  |  |  |  |
| Drinking pattern |  |  |  |  |  |  |  |  |  |  |  |  |  |  |  |  |  |  |  |  |  |  |  |  |  |  |
| Non-drinker | 228 | 64 | 32 | 17 | 10 |  |  | 557 | 109 | 59 | 40 | 19 |  |  | 1036 | 163 | 58 | 34 | 27 |  |  | 170 | 28 | 11 | 8 | 7 |
| Light (≤2 drinks/occasion) | 979 | 118 | 47 | 18 | 21 |  |  | 579 | 91 | 49 | 34 | 16 |  |  | 1628 | 162 | 56 | 33 | 30 |  |  | 565 | 69 | 32 | 17 | 5 |
| Moderate (≤4 drinks/occasion) | 899 | 101 | 41 | 18 | 11 |  |  | 1129 | 175 | 96 | 63 | 31 |  |  | 803 | 72 | 22 | 11 | 20 |  |  | 517 | 59 | 24 | 16 | 7 |
| Occasional heavy (>4 drinks/occasion <1 wk) | 1283 | 115 | 34 | 19 | 25 |  |  | 1289 | 181 | 104 | 64 | 36 |  |  | 1179 | 103 | 42 | 24 | 25 |  |  | 1807 | 157 | 57 | 37 | 31 |
| Regular heavy (>4 drinks/occasion ≥1 wk) | 284 | 30 | 12 | 5 | 8 |  |  | 573 | 90 | 40 | 26 | 29 |  |  | 157 | 15 | 7 | 6 | 2 |  |  | 143 | 7 | 2 | 1 | 3 |
| Observations | 3673 | 428 | 166 | 77 | 75 |  |  | 4127 | 646 | 348 | 227 | 131 |  |  | 4803 | 515 | 185 | 108 | 104 |  |  | 3202 | 320 | 126 | 79 | 53 |
|  |  |  |  |  |  |  |  |  |  |  |  |  |  |  |  |  |  |  |  |  |  |  |  |  |  |  |
| Binge drinking (100 g ≥1/month) |  |  |  |  |  |  |  |  |  |  |  |  |  |  |  |  |  |  |  |  |  |  |  |  |  |  |
| Non-drinker | 228 | 64 | 32 | 17 | 10 |  |  | 557 | 109 | 59 | 40 | 19 |  |  | 1036 | 163 | 58 | 34 | 27 |  |  | 170 | 28 | 11 | 8 | 7 |
| Non-binge drinker | 2711 | 292 | 111 | 47 | 47 |  |  | 2319 | 345 | 193 | 130 | 58 |  |  | 3218 | 298 | 105 | 58 | 64 |  |  | 2188 | 227 | 94 | 58 | 31 |
| Binge drinker | 734 | 72 | 23 | 13 | 18 |  |  | 1251 | 192 | 96 | 57 | 54 |  |  | 549 | 54 | 22 | 16 | 13 |  |  | 844 | 65 | 21 | 13 | 15 |
| Observations | 3673 | 428 | 166 | 77 | 75 |  |  | 4127 | 646 | 348 | 227 | 131 |  |  | 4803 | 515 | 185 | 108 | 104 |  |  | 3202 | 320 | 126 | 79 | 53 |
| Person years of follow-up (000s) |  | 29.6 |  |  |  |  |  |  | 24.6 |  |  |  |  |  |  | 33.4 |  |  |  |  |  |  | 17.3 |  |  |  |
|  |  |  |  |  |  |  |  |  |  |  |  |  |  |  |  |  |  |  |  |  |  |  |  |  |  |  |
| **Women** |  |  |  |  |  |  |  |  |  |  |  |  |  |  |  |  |  |  |  |  |  |  |  |  |  |  |
| Alcohol volume |  |  |  |  |  |  |  |  |  |  |  |  |  |  |  |  |  |  |  |  |  |  |  |  |  |  |
| Non-drinker | 736 | 80 | 36 | 15 | 7 |  |  | 892 | 77 | 52 | 28 | 4 |  |  | 2317 | 169 | 65 | 20 | 16 |  |  | 300 | 26 | 9 | 6 | 7 |
| <5 g/d | 2484 | 110 | 28 | 13 | 21 |  |  | 3770 | 174 | 84 | 50 | 25 |  |  | 2424 | 95 | 27 | 8 | 13 |  |  | 3101 | 121 | 49 | 25 | 10 |
| 5-20 g/d | 688 | 23 | 10 | 2 | 2 |  |  | 304 | 13 | 6 | 1 | 4 |  |  | 289 | 6 | 3 | 2 | 2 |  |  | 406 | 11 | 3 | 2 | 2 |
| ≥20 g/d | 195 | 15 | 4 | 0 | 4 |  |  | 45 | 1 | 0 | 0 | 0 |  |  | 58 | 4 | 1 | 1 | 2 |  |  | 45 | 3 | 1 | 1 | 0 |
| Observations | 4103 | 228 | 78 | 30 | 34 |  |  | 5011 | 265 | 142 | 79 | 33 |  |  | 5088 | 274 | 96 | 31 | 33 |  |  | 3852 | 161 | 62 | 34 | 19 |
|  |  |  |  |  |  |  |  |  |  |  |  |  |  |  |  |  |  |  |  |  |  |  |  |  |  |  |
| Drinking frequency |  |  |  |  |  |  |  |  |  |  |  |  |  |  |  |  |  |  |  |  |  |  |  |  |  |  |
| Never | 736 | 80 | 36 | 15 | 7 |  |  | 892 | 77 | 52 | 28 | 4 |  |  | 2317 | 169 | 65 | 20 | 16 |  |  | 300 | 26 | 9 | 6 | 7 |
| Never | 1147 | 58 | 19 | 7 | 12 |  |  | 2309 | 121 | 66 | 38 | 16 |  |  | 1154 | 49 | 10 | 2 | 3 |  |  | 1367 | 61 | 31 | 15 | 6 |
| <1 month | 1062 | 42 | 6 | 5 | 7 |  |  | 1394 | 54 | 20 | 12 | 10 |  |  | 1001 | 36 | 11 | 4 | 8 |  |  | 1630 | 57 | 17 | 10 | 4 |
| 1-3 month | 863 | 34 | 10 | 2 | 7 |  |  | 393 | 11 | 3 | 1 | 2 |  |  | 524 | 16 | 9 | 4 | 4 |  |  | 493 | 12 | 5 | 3 | 2 |
| 1-4 week | 295 | 14 | 7 | 1 | 1 |  |  | 23 | 2 | 1 | 0 | 1 |  |  | 92 | 4 | 1 | 1 | 2 |  |  | 62 | 5 | 0 | 0 | 0 |
| Observations | 4103 | 228 | 78 | 30 | 34 |  |  | 5011 | 265 | 142 | 79 | 33 |  |  | 5088 | 274 | 96 | 31 | 33 |  |  | 3852 | 161 | 62 | 34 | 19 |
|  |  |  |  |  |  |  |  |  |  |  |  |  |  |  |  |  |  |  |  |  |  |  |  |  |  |  |
| Drinking pattern |  |  |  |  |  |  |  |  |  |  |  |  |  |  |  |  |  |  |  |  |  |  |  |  |  |  |
| Non-drinker | 736 | 80 | 36 | 15 | 7 |  |  | 892 | 77 | 52 | 28 | 4 |  |  | 2317 | 169 | 65 | 20 | 16 |  |  | 300 | 26 | 9 | 6 | 7 |
| Light (≤0.5 drink/occasion) | 550 | 37 | 15 | 8 | 5 |  |  | 646 | 29 | 15 | 8 | 3 |  |  | 1077 | 45 | 14 | 4 | 3 |  |  | 383 | 26 | 9 | 5 | 2 |
| *Appendix Table 2 Continued* |  |  |  |  |  |  |  |  |  |  |  |  |  |  |  |  |  |  |  |  |  |  |  |  |  |  |
| Moderate (≤2 drinks/occasion) | 1584 | 64 | 19 | 5 | 10 |  |  | 2516 | 119 | 57 | 36 | 19 |  |  | 1104 | 34 | 9 | 4 | 5 |  |  | 1948 | 76 | 32 | 17 | 7 |
| Occasional heavy (>2 drinks/occasion <1 week) | 1133 | 39 | 8 | 2 | 8 |  |  | 885 | 36 | 17 | 7 | 6 |  |  | 558 | 25 | 8 | 3 | 8 |  |  | 1192 | 33 | 12 | 6 | 3 |
| Regular heavy (>2 drinks/occasion ≥1 week) | 100 | 8 | 0 | 0 | 4 |  |  | 72 | 4 | 1 | 0 | 1 |  |  | 32 | 1 | 0 | 0 | 1 |  |  | 29 | 0 | 0 | 0 | 0 |
| Observations | 4103 | 228 | 78 | 30 | 34 |  |  | 5011 | 265 | 142 | 79 | 33 |  |  | 5088 | 274 | 96 | 31 | 33 |  |  | 3852 | 161 | 62 | 34 | 19 |
|  |  |  |  |  |  |  |  |  |  |  |  |  |  |  |  |  |  |  |  |  |  |  |  |  |  |  |
| Binge drinking (60 g ≥1/month) |  |  |  |  |  |  |  |  |  |  |  |  |  |  |  |  |  |  |  |  |  |  |  |  |  |  |
| Non-drinker | 736 | 80 | 36 | 15 | 7 |  |  | 892 | 77 | 52 | 28 | 4 |  |  | 2317 | 169 | 65 | 20 | 16 |  |  | 300 | 26 | 9 | 6 | 7 |
| Non-binge drinker | 2956 | 129 | 37 | 14 | 22 |  |  | 3815 | 173 | 84 | 48 | 25 |  |  | 2623 | 98 | 27 | 10 | 15 |  |  | 3198 | 131 | 50 | 26 | 12 |
| Binge drinker | 411 | 19 | 5 | 1 | 5 |  |  | 304 | 15 | 6 | 3 | 4 |  |  | 148 | 7 | 4 | 1 | 2 |  |  | 354 | 4 | 3 | 2 | 0 |
| Observations | 4103 | 228 | 78 | 30 | 34 |  |  | 5011 | 265 | 142 | 79 | 33 |  |  | 5088 | 274 | 96 | 31 | 33 |  |  | 3852 | 161 | 62 | 34 | 19 |
| Person years of follow-up (000s) |  | 34.6 |  |  |  |  |  |  | 32.4 |  |  |  |  |  |  | 36.4 |  |  |  |  |  |  | 21.6 |  |  |  |

Standard drink = 20 g of pure alcohol.

Pre-specified alcohol-related causes of death include external causes (ICD-10 codes: F10, S00-T99, V00-Y99), liver disease (B15-19 K70-77, I85), liver cancer (C22), cancer of upper aerodigestive tract (C00-15, 32), tuberculosis(A15-19, B90), pneumonia and other relevant infectious disease (J00-39, J60-98), non-MI acute IHD (I24), non-neoplastic pancreatic disease (K85-86), and relevant ill-specified disease (R00-99) as in Zaridze et al. (14).

# Appendix Table 3. Baseline descriptive characteristics of participants by sex and center

|  |  | | |  | **Men** | | |  |  | | |  |  | | |  |  |  |  | **Women** | | |  |  | | |  |  | | |  |  | | |  |  |
| --- | --- | --- | --- | --- | --- | --- | --- | --- | --- | --- | --- | --- | --- | --- | --- | --- | --- | --- | --- | --- | --- | --- | --- | --- | --- | --- | --- | --- | --- | --- | --- | --- | --- | --- | --- | --- |
|  | **Czech towns** | | |  | **Novosibirsk** | | |  | **Krakow** | | |  | **Kaunas** | | |  |  |  |  | **Czech towns** | | |  | **Novosibirsk** | | |  | **Krakow** | | |  | **Kaunas** | | |  |  |
|  | **N** | **Mean** | **SD** |  | **N** | **Mean** | **SD** |  | **N** | **Mean** | **SD** |  | **N** | **Mean** | **SD** |  | **Total** |  |  | **N** | **Mean** | **SD** |  | **N** | **Mean** | **SD** |  | **N** | **Mean** | **SD** |  | **N** | **Mean** | **SD** |  | **Total** |
| Age | 3772 | 58.5 | 7.1 |  | 4128 | 58.2 | 7.0 |  | 4846 | 57.9 | 7.0 |  | 3243 | 61.1 | 7.6 |  | 3311.7 |  |  | 4280 | 57.9 | 7.1 |  | 5011 | 58.0 | 7.1 |  | 5125 | 57.4 | 7.0 |  | 3899 | 60.9 | 7.6 |  | 18299 |
| Household assets | 3704 | 6.7 | 2.0 |  | 4098 | 5.3 | 2.0 |  | 4804 | 6.5 | 2.1 |  | 3175 | 7.0 | 2.0 |  | 15753 |  |  | 4219 | 6.2 | 2.0 |  | 4998 | 4.8 | 1.9 |  | 5079 | 6.0 | 2.1 |  | 3833 | 6.3 | 2.0 |  | 18113 |
| Education |  |  |  |  |  |  |  |  |  |  |  |  |  |  |  |  |  |  |  |  |  |  |  |  |  |  |  |  |  |  |  |  |  |  |  |  |
| Primary | 227 | 6.1 |  |  | 468 | 11.3 |  |  | 447 | 9.2 |  |  | 429 | 13.4 |  |  | 1571 |  |  | 765 | 17.9 |  |  | 477 | 9.5 |  |  | 679 | 13.3 |  |  | 458 | 11.9 |  |  | 2379 |
| Secondary | 2843 | 75.8 |  |  | 2330 | 56.4 |  |  | 2936 | 60.6 |  |  | 1697 | 53.0 |  |  | 9806 |  |  | 3069 | 72.0 |  |  | 3211 | 64.1 |  |  | 3053 | 59.6 |  |  | 2164 | 56.1 |  |  | 11497 |
| University | 679 | 18.1 |  |  | 1330 | 32.2 |  |  | 1460 | 30.1 |  |  | 1078 | 33.6 |  |  | 4547 |  |  | 428 | 10.0 |  |  | 1323 | 26.4 |  |  | 1388 | 27.1 |  |  | 1232 | 32.0 |  |  | 4371 |
| Current employment status |  |  |  |  |  |  |  |  |  |  |  |  |  |  |  |  |  |  |  |  |  |  |  |  |  |  |  |  |  |  |  |  |  |  |  |  |
| Employed/self-employed | 1851 | 49.7 |  |  | 1585 | 38.4 |  |  | 1901 | 39.4 |  |  | 1323 | 41.5 |  |  | 6660 |  |  | 1672 | 39.5 |  |  | 1486 | 29.7 |  |  | 1689 | 33.0 |  |  | 1275 | 33.3 |  |  | 6122 |
| Pensionable age, still working | 310 | 8.3 |  |  | 878 | 21.3 |  |  | 369 | 7.6 |  |  | 655 | 20.6 |  |  | 2212 |  |  | 332 | 7.8 |  |  | 816 | 16.3 |  |  | 289 | 5.7 |  |  | 574 | 15.0 |  |  | 2011 |
| Pensionable age, not working | 1433 | 38.5 |  |  | 1343 | 32.6 |  |  | 2206 | 45.7 |  |  | 947 | 29.7 |  |  | 5929 |  |  | 2060 | 48.6 |  |  | 2426 | 48.5 |  |  | 2731 | 53.4 |  |  | 1664 | 43.4 |  |  | 8881 |
| Unemployed | 112 | 3.0 |  |  | 210 | 5.1 |  |  | 266 | 5.5 |  |  | 107 | 3.4 |  |  | 695 |  |  | 113 | 2.7 |  |  | 129 | 2.6 |  |  | 186 | 3.6 |  |  | 149 | 3.9 |  |  | 577 |
| Other | 17 | 0.5 |  |  | 109 | 2.6 |  |  | 84 | 1.7 |  |  | 154 | 4.8 |  |  | 364 |  |  | 61 | 1.4 |  |  | 150 | 3.0 |  |  | 219 | 4.3 |  |  | 168 | 4.4 |  |  | 598 |
| Marital status |  |  |  |  |  |  |  |  |  |  |  |  |  |  |  |  |  |  |  |  |  |  |  |  |  |  |  |  |  |  |  |  |  |  |  |  |
| Partnered | 3166 | 84.3 |  |  | 3626 | 87.8 |  |  | 4189 | 86.7 |  |  | 2723 | 85.0 |  |  | 13704 |  |  | 2909 | 68.3 |  |  | 2979 | 59.4 |  |  | 3416 | 66.8 |  |  | 2197 | 57.0 |  |  | 11501 |
| Not partnered | 589 | 15.7 |  |  | 502 | 12.2 |  |  | 643 | 13.3 |  |  | 481 | 15.0 |  |  | 2215 |  |  | 1353 | 31.7 |  |  | 2032 | 40.6 |  |  | 1700 | 33.2 |  |  | 1657 | 43.0 |  |  | 6742 |
| Smoking status |  |  |  |  |  |  |  |  |  |  |  |  |  |  |  |  |  |  |  |  |  |  |  |  |  |  |  |  |  |  |  |  |  |  |  |  |
| Never smoker | 1190 | 31.9 |  |  | 1066 | 26.0 |  |  | 1345 | 27.9 |  |  | 1228 | 38.9 |  |  | 4829 |  |  | 2291 | 54.1 |  |  | 4281 | 86.1 |  |  | 2574 | 50.6 |  |  | 3202 | 84.0 |  |  | 12348 |
| Ex-smoker | 1423 | 38.1 |  |  | 1016 | 24.8 |  |  | 1747 | 36.3 |  |  | 994 | 31.5 |  |  | 5180 |  |  | 927 | 21.9 |  |  | 217 | 4.4 |  |  | 1071 | 21.0 |  |  | 274 | 7.2 |  |  | 2489 |
| ≤10 cigs/d | 402 | 10.8 |  |  | 524 | 12.8 |  |  | 457 | 9.5 |  |  | 338 | 10.7 |  |  | 1721 |  |  | 608 | 14.4 |  |  | 365 | 7.3 |  |  | 623 | 12.2 |  |  | 262 | 6.9 |  |  | 1858 |
| 11-20 cigs/d | 582 | 15.6 |  |  | 1191 | 29.1 |  |  | 900 | 18.7 |  |  | 507 | 16.1 |  |  | 3180 |  |  | 382 | 9.0 |  |  | 102 | 2.1 |  |  | 700 | 13.8 |  |  | 70 | 1.8 |  |  | 1254 |
| ≥21 cigs/d | 134 | 3.6 |  |  | 299 | 7.3 |  |  | 364 | 7.6 |  |  | 90 | 2.9 |  |  | 887 |  |  | 27 | 0.6 |  |  | 7 | 0.1 |  |  | 121 | 2.4 |  |  | 5 | 0.1 |  |  | 160 |
| Leisure-time physical activity |  |  |  |  |  |  |  |  |  |  |  |  |  |  |  |  |  |  |  |  |  |  |  |  |  |  |  |  |  |  |  |  |  |  |  |  |
| None | 1165 | 31.8 |  |  | 2910 | 70.5 |  |  | 1326 | 28.9 |  |  | 1505 | 50.1 |  |  | 6906 |  |  | 1222 | 29.6 |  |  | 3642 | 72.8 |  |  | 1483 | 30.5 |  |  | 1844 | 50.3 |  |  | 8191 |
| Some | 2503 | 68.2 |  |  | 1217 | 29.5 |  |  | 3263 | 71.1 |  |  | 1501 | 49.9 |  |  | 8484 |  |  | 2902 | 70.4 |  |  | 1363 | 27.2 |  |  | 3374 | 69.5 |  |  | 1824 | 49.7 |  |  | 9463 |
| BMI | 3134 | 28.3 | 4.0 |  | 4128 | 26.6 | 4.4 |  | 4258 | 28.0 | 4.0 |  | 3220 | 28.5 | 4.6 |  | 14711 |  |  | 3672 | 28.1 | 5.0 |  | 5011 | 30.2 | 5.7 |  | 5116 | 28.3 | 5.1 |  | 3866 | 30.1 | 5.7 |  | 17049 |
| Self-reported CVD |  |  |  |  |  |  |  |  |  |  |  |  |  |  |  |  |  |  |  |  |  |  |  |  |  |  |  |  |  |  |  |  |  |  |  |  |
| No | 3039 | 84.4 |  |  | 3159 | 76.5 |  |  | 3657 | 76.4 |  |  | 2620 | 81.8 |  |  | 12475 |  |  | 3627 | 90.4 |  |  | 4009 | 80.0 |  |  | 3997 | 78.9 |  |  | 3153 | 81.8 |  |  | 14786 |
| Yes | 563 | 15.6 |  |  | 969 | 23.5 |  |  | 1128 | 23.6 |  |  | 582 | 18.2 |  |  | 3243 |  |  | 383 | 9.6 |  |  | 1002 | 20.0 |  |  | 1068 | 21.1 |  |  | 700 | 18.2 |  |  | 3153 |
| Self-reported cancer |  |  |  |  |  |  |  |  |  |  |  |  |  |  |  |  |  |  |  |  |  |  |  |  |  |  |  |  |  |  |  |  |  |  |  |  |
| No | 3470 | 95.8 |  |  | 4073 | 98.7 |  |  | 4643 | 96.7 |  |  | 3038 | 94.8 |  |  | 15224 |  |  | 3711 | 91.7 |  |  | 4812 | 96.0 |  |  | 4774 | 93.9 |  |  | 3494 | 90.7 |  |  | 16791 |
| Yes | 153 | 4.2 |  |  | 55 | 1.3 |  |  | 157 | 3.3 |  |  | 166 | 5.2 |  |  | 531 |  |  | 335 | 8.3 |  |  | 199 | 4.0 |  |  | 312 | 6.1 |  |  | 358 | 9.3 |  |  | 1204 |
| Depressive symptoms |  |  |  |  |  |  |  |  |  |  |  |  |  |  |  |  |  |  |  |  |  |  |  |  |  |  |  |  |  |  |  |  |  |  |  |  |
| No | 2987 | 82.0 |  |  | 3376 | 81.8 |  |  | 3635 | 75.6 |  |  | 2623 | 81.9 |  |  | 12621 |  |  | 2986 | 72.1 |  |  | 3123 | 62.3 |  |  | 3091 | 60.8 |  |  | 2528 | 65.6 |  |  | 11728 |
| Yes | 655 | 18.0 |  |  | 750 | 18.2 |  |  | 1175 | 24.4 |  |  | 579 | 18.1 |  |  | 3159 |  |  | 1158 | 27.9 |  |  | 1888 | 37.7 |  |  | 1994 | 39.2 |  |  | 1323 | 34.4 |  |  | 6363 |

# **Appendix Table 4. Associations of baseline graduated frequency-based alcohol indices with other and repeated alcohol measures and serum GGT levels**

|  | **Weekly alcohol**  **consumption (g/d)** | | | |  | **FFQ-based**  **alcohol volume (g/d)** | | | |  | **Wave 2 GFQ-based**  **alcohol volume (g/d)** | | | |  | **Novosibirsk**  **GGT** | | | |  | **Czech, Krakow &Kaunas**  **GGT** | | | |
| --- | --- | --- | --- | --- | --- | --- | --- | --- | --- | --- | --- | --- | --- | --- | --- | --- | --- | --- | --- | --- | --- | --- | --- | --- |
|  | **N** | **Mean** | **SD** | **Median** |  | **N** | **Mean** | **SD** | **Median** |  | **N** | **Mean** | **SD** | **Median** |  | **N** | **Mean** | **SD** | **Median** |  | **N** | **Mean** | **SD** | **Median** |
| ***Baseline GFQ-based alcohol measures*** |  |  |  |  |  |  |  |  |  |  |  |  |  |  |  |  |  |  |  |  |  |  |  |  |
| **Men** |  |  |  |  |  |  |  |  |  |  |  |  |  |  |  |  |  |  |  |  |  |  |  |  |
| Alcohol volume |  |  |  |  |  |  |  |  |  |  |  |  |  |  |  |  |  |  |  |  |  |  |  |  |
| Non-drinker | 1976 | 0.5 | 4.6 | 0.0 |  | 1648 | 0.5 | 4.5 | 0.0 |  | 1068 | 3.6 | 10.5 | 0.3 |  | 549 | 30.0 | 35.5 | 23.0 |  | 462 | 32.3 | 56.8 | 18.4 |
| <10 g/d | 8615 | 10.3 | 21.4 | 2.9 |  | 6056 | 5.6 | 10.4 | 2.6 |  | 4356 | 8.2 | 13.6 | 4.5 |  | 2009 | 36.2 | 43.3 | 27.0 |  | 2037 | 34.9 | 62.4 | 18.5 |
| 10-60 g/d | 4668 | 30.1 | 31.7 | 21.7 |  | 3453 | 18.4 | 18.4 | 11.3 |  | 2390 | 21.7 | 24.6 | 16.0 |  | 1414 | 45.7 | 57.0 | 31.0 |  | 1030 | 48.2 | 83.8 | 23.4 |
| ≥60 g/d | 441 | 78.9 | 61.8 | 67.4 |  | 342 | 40.7 | 31.9 | 35.9 |  | 224 | 47.6 | 40.5 | 36.3 |  | 93 | 63.4 | 61.4 | 44.0 |  | 106 | 91.2 | 127.8 | 38.9 |
| Total | 15700 | 16.9 | 29.6 | 5.7 |  | 11499 | 9.8 | 16.3 | 4.3 |  | 8038 | 12.7 | 20.5 | 5.3 |  | 4065 | 39.3 | 48.5 | 28.0 |  | 3635 | 40.0 | 71.9 | 20.2 |
|  |  |  |  |  |  |  |  |  |  |  |  |  |  |  |  |  |  |  |  |  |  |  |  |  |
| Drinking frequency |  |  |  |  |  |  |  |  |  |  |  |  |  |  |  |  |  |  |  |  |  |  |  |  |
| Never | 1976 | 0.5 | 4.6 | 0.0 |  | 1648 | 0.5 | 4.5 | 0.0 |  | 1068 | 3.6 | 10.5 | 0.3 |  | 549 | 30.0 | 35.5 | 23.0 |  | 462 | 32.3 | 56.8 | 18.4 |
| <1 month | 2204 | 6.0 | 17.9 | 0.0 |  | 1585 | 3.4 | 9.3 | 0.6 |  | 1139 | 6.2 | 12.0 | 2.1 |  | 561 | 32.1 | 27.1 | 26.0 |  | 531 | 31.1 | 53.5 | 17.1 |
| 1-3 month | 3868 | 9.4 | 20.1 | 2.6 |  | 2602 | 5.5 | 10.7 | 2.0 |  | 1814 | 7.8 | 14.1 | 3.4 |  | 1047 | 37.1 | 46.7 | 27.0 |  | 859 | 33.8 | 58.5 | 18.0 |
| 1-4 week | 5246 | 19.9 | 26.4 | 11.4 |  | 3782 | 11.7 | 15.5 | 7.0 |  | 2684 | 14.3 | 19.1 | 7.2 |  | 1564 | 43.0 | 47.3 | 31.0 |  | 1075 | 40.7 | 70.9 | 21.0 |
| 5+ week | 2406 | 45.8 | 44.3 | 34.3 |  | 1882 | 25.3 | 22.4 | 18.7 |  | 1333 | 28.9 | 30.3 | 21.4 |  | 344 | 55.5 | 85.0 | 35.0 |  | 708 | 58.1 | 100.2 | 26.5 |
| Total | 15700 | 16.9 | 29.6 | 5.7 |  | 11499 | 9.8 | 16.3 | 4.3 |  | 8038 | 12.7 | 20.5 | 5.3 |  | 4065 | 39.3 | 48.5 | 28.0 |  | 3635 | 40.0 | 71.9 | 20.2 |
|  |  |  |  |  |  |  |  |  |  |  |  |  |  |  |  |  |  |  |  |  |  |  |  |  |
| Drinking pattern |  |  |  |  |  |  |  |  |  |  |  |  |  |  |  |  |  |  |  |  |  |  |  |  |
| Non-drinker | 1976 | 0.5 | 4.6 | 0.0 |  | 1648 | 0.5 | 4.5 | 0.0 |  | 1068 | 3.6 | 10.5 | 0.3 |  | 549 | 30.0 | 35.5 | 23.0 |  | 462 | 32.3 | 56.8 | 18.4 |
| Light (≤2 drinks/occasion) | 3716 | 12.3 | 23.8 | 4.0 |  | 2809 | 5.6 | 10.0 | 1.9 |  | 2143 | 8.7 | 15.0 | 3.9 |  | 570 | 35.2 | 45.9 | 26.0 |  | 1072 | 34.7 | 59.0 | 19.1 |
| Moderate (≤4 drinks/occasion) | 3330 | 15.8 | 25.4 | 7.7 |  | 2631 | 10.2 | 14.7 | 4.9 |  | 1796 | 12.1 | 18.4 | 5.5 |  | 1114 | 38.2 | 43.9 | 28.0 |  | 758 | 40.0 | 72.1 | 19.4 |
| Occasional heavy (>4 drinks/occasion <1 wk) | 5531 | 19.7 | 28.4 | 11.4 |  | 3495 | 12.8 | 17.0 | 6.8 |  | 2412 | 15.9 | 21.0 | 8.0 |  | 1269 | 39.6 | 41.0 | 29.0 |  | 1187 | 42.2 | 77.5 | 21.1 |
| Regular heavy (>4 drinks/occasion ≥1 wk) | 1147 | 49.7 | 52.3 | 31.4 |  | 916 | 26.2 | 27.2 | 14.8 |  | 619 | 31.5 | 34.8 | 19.3 |  | 563 | 53.8 | 75.6 | 34.0 |  | 156 | 82.6 | 117.9 | 37.3 |
| Total | 15700 | 16.9 | 29.6 | 5.7 |  | 11499 | 9.8 | 16.3 | 4.3 |  | 8038 | 12.7 | 20.5 | 5.3 |  | 4065 | 39.3 | 48.5 | 28.0 |  | 3635 | 40.0 | 71.9 | 20.2 |
|  |  |  |  |  |  |  |  |  |  |  |  |  |  |  |  |  |  |  |  |  |  |  |  |  |
| Binge drinking (100 g ≥1/month) |  |  |  |  |  |  |  |  |  |  |  |  |  |  |  |  |  |  |  |  |  |  |  |  |
| Non-drinker | 1976 | 0.5 | 4.6 | 0.0 |  | 1648 | 0.5 | 4.5 | 0.0 |  | 1068 | 3.6 | 10.5 | 0.3 |  | 549 | 30.0 | 35.5 | 23.0 |  | 462 | 32.3 | 56.8 | 18.4 |
| Non-binge | 10369 | 14.9 | 24.9 | 5.7 |  | 7520 | 8.8 | 13.6 | 4.3 |  | 5448 | 11.1 | 17.1 | 5.3 |  | 2288 | 36.9 | 40.9 | 27.0 |  | 2596 | 37.6 | 68.1 | 19.3 |
| Binge | 3355 | 32.6 | 41.9 | 21.1 |  | 2331 | 19.5 | 23.1 | 10.0 |  | 1522 | 24.6 | 29.8 | 15.9 |  | 1228 | 47.8 | 63.2 | 32.0 |  | 577 | 57.1 | 93.7 | 26.7 |
| Total | 15700 | 16.9 | 29.6 | 5.7 |  | 11499 | 9.8 | 16.3 | 4.3 |  | 8038 | 12.7 | 20.5 | 5.3 |  | 4065 | 39.3 | 48.5 | 28.0 |  | 3635 | 40.0 | 71.9 | 20.2 |
|  |  |  |  |  |  |  |  |  |  |  |  |  |  |  |  |  |  |  |  |  |  |  |  |  |
| **Women** |  |  |  |  |  |  |  |  |  |  |  |  |  |  |  |  |  |  |  |  |  |  |  |  |
| Alcohol volume |  |  |  |  |  |  |  |  |  |  |  |  |  |  |  |  |  |  |  |  |  |  |  |  |
| Non-drinker | 4206 | 0.2 | 3.9 | 0.0 |  | 3537 | 0.3 | 3.4 | 0.0 |  | 2413 | 0.9 | 2.7 | 0.1 |  | 882 | 27.3 | 21.4 | 22.0 |  | 508 | 26.7 | 56.1 | 12.9 |
| <5 g/d | 11631 | 2.5 | 9.5 | 0.0 |  | 8113 | 1.6 | 3.6 | 0.6 |  | 6033 | 2.3 | 6.1 | 0.6 |  | 3734 | 28.9 | 26.8 | 22.0 |  | 949 | 23.1 | 45.2 | 10.9 |
| 5-20 g/d | 1678 | 11.5 | 19.1 | 5.8 |  | 1192 | 6.2 | 7.0 | 4.3 |  | 866 | 7.2 | 10.9 | 5.3 |  | 298 | 33.0 | 32.0 | 25.0 |  | 149 | 44.1 | 110.3 | 12.5 |
| ≥20 g/d | 339 | 26.5 | 32.0 | 17.1 |  | 268 | 15.1 | 14.3 | 10.0 |  | 183 | 15.6 | 21.4 | 10.9 |  | 45 | 34.9 | 25.5 | 27.0 |  | 41 | 79.9 | 167.6 | 19.5 |
| Total | 17854 | 3.3 | 11.6 | 0.0 |  | 13110 | 1.9 | 5.1 | 0.6 |  | 9495 | 2.6 | 7.2 | 0.6 |  | 4959 | 28.9 | 26.3 | 22.0 |  | 1647 | 27.5 | 63.5 | 11.9 |
|  |  |  |  |  |  |  |  |  |  |  |  |  |  |  |  |  |  |  |  |  |  |  |  |  |
| Drinking frequency |  |  |  |  |  |  |  |  |  |  |  |  |  |  |  |  |  |  |  |  |  |  |  |  |
| Never | 4206 | 0.2 | 3.9 | 0.0 |  | 3537 | 0.3 | 3.4 | 0.0 |  | 2413 | 0.9 | 2.7 | 0.1 |  | 882 | 27.3 | 21.4 | 22.0 |  | 508 | 26.7 | 56.1 | 12.9 |
| <1 month | 5892 | 1.3 | 6.5 | 0.0 |  | 4291 | 1.0 | 2.7 | 0.6 |  | 3137 | 1.4 | 3.2 | 0.3 |  | 2282 | 29.1 | 29.5 | 22.0 |  | 477 | 25.7 | 51.1 | 11.6 |
| 1-3 month | 5028 | 3.1 | 11.0 | 0.0 |  | 3251 | 2.0 | 4.3 | 1.2 |  | 2440 | 2.9 | 8.1 | 0.8 |  | 1383 | 28.7 | 22.6 | 22.0 |  | 389 | 20.7 | 40.5 | 10.4 |
| 1-4 week | 2259 | 10.2 | 16.8 | 5.7 |  | 1651 | 4.9 | 6.0 | 3.7 |  | 1239 | 6.0 | 10.0 | 2.3 |  | 389 | 31.7 | 29.1 | 24.0 |  | 218 | 35.5 | 89.9 | 12.5 |
| 5+ week | 469 | 23.9 | 32.0 | 15.4 |  | 380 | 14.3 | 12.7 | 10.0 |  | 266 | 14.6 | 18.2 | 12.5 |  | 23 | 33.8 | 23.0 | 27.0 |  | 55 | 68.0 | 153.8 | 11.4 |
| Total | 17854 | 3.3 | 11.6 | 0.0 |  | 13110 | 1.9 | 5.1 | 0.6 |  | 9495 | 2.6 | 7.2 | 0.6 |  | 4959 | 28.9 | 26.3 | 22.0 |  | 1647 | 27.5 | 63.5 | 11.9 |
|  |  |  |  |  |  |  |  |  |  |  |  |  |  |  |  |  |  |  |  |  |  |  |  |  |
| Drinking pattern |  |  |  |  |  |  |  |  |  |  |  |  |  |  |  |  |  |  |  |  |  |  |  |  |
| Non-drinker | 4206 | 0.2 | 3.9 | 0.0 |  | 3537 | 0.3 | 3.4 | 0.0 |  | 2413 | 0.9 | 2.7 | 0.1 |  | 882 | 27.3 | 21.4 | 22.0 |  | 508 | 26.7 | 56.1 | 12.9 |
| Light (≤0.5 drink/occasion) | 2586 | 2.8 | 10.3 | 0.0 |  | 2065 | 1.2 | 3.3 | 0.6 |  | 1564 | 2.2 | 6.2 | 0.6 |  | 638 | 27.8 | 22.3 | 22.0 |  | 257 | 26.6 | 57.4 | 12.1 |
| Moderate (≤2 drinks/occasion) | 7092 | 2.7 | 8.4 | 0.0 |  | 4905 | 2.1 | 4.7 | 0.6 |  | 3636 | 2.5 | 6.2 | 0.6 |  | 2489 | 28.3 | 24.2 | 22.0 |  | 584 | 25.6 | 55.5 | 11.2 |
| Occasional heavy (>2 drinks/occasion <1 week) | 3740 | 6.9 | 17.9 | 2.6 |  | 2427 | 4.0 | 6.7 | 1.8 |  | 1755 | 4.8 | 9.4 | 2.1 |  | 879 | 32.3 | 35.4 | 24.0 |  | 279 | 28.6 | 78.4 | 9.6 |
| *Appendix Table 4 Continued* |  |  |  |  |  |  |  |  |  |  |  |  |  |  |  |  |  |  |  |  |  |  |  |  |
| Regular heavy (>2 drinks/occasion ≥1 week) | 230 | 22.2 | 30.3 | 13.4 |  | 176 | 10.0 | 12.8 | 5.3 |  | 127 | 14.0 | 25.6 | 5.3 |  | 71 | 37.9 | 43.6 | 27.0 |  | 19 | 104.7 | 177.8 | 31.5 |
| Total | 17854 | 3.3 | 11.6 | 0.0 |  | 13110 | 1.9 | 5.1 | 0.6 |  | 9495 | 2.6 | 7.2 | 0.6 |  | 4959 | 28.9 | 26.3 | 22.0 |  | 1647 | 27.5 | 63.5 | 11.9 |
|  |  |  |  |  |  |  |  |  |  |  |  |  |  |  |  |  |  |  |  |  |  |  |  |  |
| Binge drinking (60 g ≥1/month) |  |  |  |  |  |  |  |  |  |  |  |  |  |  |  |  |  |  |  |  |  |  |  |  |
| Non-drinker | 4206 | 0.2 | 3.9 | 0.0 |  | 3537 | 0.3 | 3.4 | 0.0 |  | 2413 | 0.9 | 2.7 | 0.1 |  | 882 | 27.3 | 21.4 | 22.0 |  | 508 | 26.7 | 56.1 | 12.9 |
| Non-binge | 12442 | 3.4 | 11.3 | 0.0 |  | 8777 | 2.2 | 4.7 | 0.6 |  | 6535 | 2.8 | 7.0 | 0.6 |  | 3776 | 28.9 | 26.9 | 22.0 |  | 1045 | 25.5 | 57.0 | 11.0 |
| Binge | 1206 | 12.3 | 22.8 | 5.7 |  | 796 | 6.5 | 9.8 | 3.2 |  | 547 | 8.3 | 15.4 | 3.7 |  | 301 | 33.8 | 31.1 | 25.0 |  | 94 | 54.2 | 129.8 | 15.3 |
| Total | 17854 | 3.3 | 11.6 | 0.0 |  | 13110 | 1.9 | 5.1 | 0.6 |  | 9495 | 2.6 | 7.2 | 0.6 |  | 4959 | 28.9 | 26.3 | 22.0 |  | 1647 | 27.5 | 63.5 | 11.9 |

Separate questions on typical weekly intake of wine, beer and spirits were asked at baseline in all cohorts and used to calculate typical weekly alcohol intake. Baseline alcohol intake calculated from the food frequency questionnaire (FFQ) and repeated measures of alcohol consumption were not available in Kaunas. FFQ was administered separately at baseline in all other centers, and alcohol intake was derived independently. GGT was assessed in all Novosibirsk participants and a subsample of participants in the other three centers. GGT was analyzed in a local laboratory in Novosibirsk and in Laboratory for Health Protection Research (GBO)/National Institute for Public Health and the Environment (RIVM), Bilthoven, The Netherlands for the remaining centers.

#

# Appendix Table 5. Cox regression results for alcohol and non-CHD cardiovascular and cerebral stroke deaths

|  | **Men** | | | | | | |  | **Women** | | | | | | |
| --- | --- | --- | --- | --- | --- | --- | --- | --- | --- | --- | --- | --- | --- | --- | --- |
|  | **Age-adjusted** | | |  | **Fully-adjusted^1^** | | |  | **Age-adjusted** | | |  | **Fully-adjusted^1^** | | |
| **Non-CHD CVD deaths** | **HR** | **95% CI** | |  | **HR** | **95% CI** | |  | **HR** | **95% CI** | |  | **HR** | **95% CI** | |
| Alcohol intake |  |  |  |  |  |  |  |  |  |  |  |  |  |  |  |
| Non-drinker | 1.45 | [1.08 | 1.95] |  | 1.18 | [0.88 | 1.59] |  | 2.12 | [1.56 | 2.89] |  | 1.73 | [1.27 | 2.36] |
| <5/10 g/d | 1.00 | . | . |  | 1.00 | . | . |  | 1.00 | . | . |  | 1.00 | . | . |
| 5-20/10-60 g/d | 0.77 | [0.58 | 1.01] |  | 0.78 | [0.59 | 1.04] |  | 1.50 | [0.86 | 2.62] |  | 1.48 | [0.84 | 2.59] |
| ≥20/60 g/d | 1.36 | [0.73 | 2.51] |  | 1.08 | [0.58 | 2.02] |  | 2.21 | [0.80 | 6.09] |  | 2.02 | [0.73 | 5.60] |
|  |  |  |  |  |  |  |  |  |  |  |  |  |  |  |  |
| Drinking frequency |  |  |  |  |  |  |  |  |  |  |  |  |  |  |  |
| Never | 1.49 | [1.02 | 2.17] |  | 1.25 | [0.86 | 1.82] |  | 1.78 | [1.26 | 2.49] |  | 1.50 | [1.07 | 2.11] |
| <1 month | 1.00 | . | . |  | 1.00 | . | . |  | 1.00 | . | . |  | 1.00 | . | . |
| 1-3 month | 1.06 | [0.75 | 1.49] |  | 1.08 | [0.76 | 1.52] |  | 0.59 | [0.36 | 0.94] |  | 0.62 | [0.38 | 1.00] |
| 1-4 week | 0.87 | [0.62 | 1.22] |  | 0.94 | [0.67 | 1.32] |  | 1.02 | [0.59 | 1.77] |  | 1.14 | [0.66 | 1.98] |
| 5+ week | 0.93 | [0.61 | 1.40] |  | 0.95 | [0.63 | 1.44] |  | 1.80 | [0.81 | 4.01] |  | 1.70 | [0.76 | 3.81] |
|  |  |  |  |  |  |  |  |  |  |  |  |  |  |  |  |
| Drinking pattern |  |  |  |  |  |  |  |  |  |  |  |  |  |  |  |
| Non-drinker | 1.55 | [1.10 | 2.17] |  | 1.27 | [0.91 | 1.78] |  | 1.89 | [1.23 | 2.90] |  | 1.58 | [1.03 | 2.43] |
| Light drinker | 1.00 | . | . |  | 1.00 | . | . |  | 1.00 | . | . |  | 1.00 | . | . |
| Moderate drinker | 1.02 | [0.74 | 1.40] |  | 1.05 | [0.77 | 1.45] |  | 0.85 | [0.53 | 1.34] |  | 0.90 | [0.57 | 1.43] |
| Occasional heavy | 0.97 | [0.72 | 1.32] |  | 1.01 | [0.74 | 1.38] |  | 1.15 | [0.66 | 1.98] |  | 1.07 | [0.62 | 1.86] |
| Regular heavy drinker | 1.03 | [0.64 | 1.66] |  | 0.97 | [0.60 | 1.57] |  | 0.84 | [0.11 | 6.11] |  | 0.65 | [0.09 | 4.78] |
|  |  |  |  |  |  |  |  |  |  |  |  |  |  |  |  |
| Binge drinking |  |  |  |  |  |  |  |  |  |  |  |  |  |  |  |
| Non-drinker | 1.58 | [1.18 | 2.12] |  | 1.27 | [0.94 | 1.70] |  | 2.10 | [1.55 | 2.85] |  | 1.71 | [1.26 | 2.33] |
| Non-binge drinker | 1.00 | . | . |  | 1.00 | . | . |  | 1.00 | . | . |  | 1.00 | . | . |
| Binge drinker | 1.10 | [0.83 | 1.46] |  | 1.03 | [0.77 | 1.38] |  | 1.84 | [0.98 | 3.46] |  | 1.54 | [0.81 | 2.90] |
| N/ Person yrs (000s)/ N. of deaths | 15989 | 105.2 | 340 |  | 15989 | 105.2 | 340 |  | 18315 | 125.0 | 210 |  | 18315 | 125.0 | 210 |
|  |  |  |  |  |  |  |  |  |  |  |  |  |  |  |  |
| **Cerebral stroke deaths** |  |  |  |  |  |  |  |  |  |  |  |  |  |  |  |
| Alcohol intake |  |  |  |  |  |  |  |  |  |  |  |  |  |  |  |
| Non-drinker | 1.15 | 0.58 | 2.26 |  | 1.01 | 0.51 | 2.00 |  | 0.84 | 0.34 | 2.10 |  | 0.66 | 0.26 | 1.67 |
| <5/10 g/d | 1.00 | . | . |  | 1.00 | . | . |  | 1.00 | . | . |  | 1.00 | . | . |
| 5-20/10-60 g/d | 0.75 | 0.41 | 1.39 |  | 0.77 | 0.41 | 1.44 |  | 0.58 | 0.08 | 4.36 |  | 0.68 | 0.09 | 5.15 |
| ≥20/60 g/d | 1.78 | 0.43 | 7.44 |  | 1.74 | 0.41 | 7.36 |  | 3.65 | 0.48 | 28.08 |  | 3.78 | 0.47 | 30.41 |
|  |  |  |  |  |  |  |  |  |  |  |  |  |  |  |  |
| Drinking frequency |  |  |  |  |  |  |  |  |  |  |  |  |  |  |  |
| Never | 1.56 | 0.64 | 3.80 |  | 1.44 | 0.59 | 3.52 |  | 0.66 | 0.26 | 1.69 |  | 0.56 | 0.22 | 1.44 |
| <1 month | 1.00 | . | . |  | 1.00 | . | . |  | 1.00 | . | . |  | 1.00 | . | . |
| 1-3 month | 1.62 | 0.75 | 3.51 |  | 1.73 | 0.79 | 3.77 |  | 0.31 | 0.09 | 1.07 |  | 0.36 | 0.10 | 1.26 |
| 1-4 week | 0.96 | 0.42 | 2.18 |  | 1.04 | 0.45 | 2.39 |  | 0.68 | 0.15 | 3.05 |  | 0.88 | 0.19 | 4.03 |
| 5+ week | 1.95 | 0.76 | 4.99 |  | 2.18 | 0.84 | 5.65 |  | 1.57 | 0.20 | 12.46 |  | 1.90 | 0.23 | 15.68 |
|  |  |  |  |  |  |  |  |  |  |  |  |  |  |  |  |
| Drinking pattern |  |  |  |  |  |  |  |  |  |  |  |  |  |  |  |
| Non-drinker | 1.32 | 0.60 | 2.94 |  | 1.23 | 0.55 | 2.75 |  | 1.14 | 0.33 | 3.97 |  | 0.94 | 0.27 | 3.29 |
| Light drinker | 1.00 | . | . |  | 1.00 | . | . |  | 1.00 | . | . |  | 1.00 | . | . |
| Moderate drinker | 1.38 | 0.69 | 2.76 |  | 1.54 | 0.77 | 3.08 |  | 1.56 | 0.52 | 4.73 |  | 1.70 | 0.56 | 5.18 |
| Occasional heavy | 0.92 | 0.44 | 1.89 |  | 1.02 | 0.49 | 2.13 |  | 0.61 | 0.11 | 3.38 |  | 0.64 | 0.11 | 3.60 |
| Regular heavy drinker | 1.01 | 0.35 | 2.87 |  | 1.07 | 0.37 | 3.09 |  | 6.85 | 0.74 | 63.43 |  | 6.38 | 0.64 | 63.40 |
|  |  |  |  |  |  |  |  |  |  |  |  |  |  |  |  |
| Binge drinking |  |  |  |  |  |  |  |  |  |  |  |  |  |  |  |
| Non-drinker | 1.15 | 0.59 | 2.24 |  | 1.00 | 0.51 | 1.96 |  | 0.91 | 0.36 | 2.27 |  | 0.71 | 0.28 | 1.80 |
| Non-binge drinker | 1.00 | . | . |  | 1.00 | . | . |  | 1.00 | . | . |  | 1.00 | . | . |
| Binge drinker | 0.73 | 0.37 | 1.42 |  | 0.73 | 0.37 | 1.44 |  | 1.95 | 0.45 | 8.51 |  | 2.05 | 0.46 | 9.12 |
| N/ Person yrs (000s)/ N. of deaths | 1598 | 68 | 105.2 |  | 15989 | 68 | 105.2 |  | 18315 | 31.00 | 125.0 |  | 18315.00 | 31 | 125.0 |

^1^Adjusted for age, education, marital status, economic activity, asset score, subjective hardship score, smoking, physical activity, BMI, prevalent CVD and cancer, and depressive symptoms.

In men, daily alcohol intake was not associated with non-CHD cardiovascular deaths. The adjusted HRs for non-CHD cardiovascular deaths (<40% of all CVD deaths in men and 54% in women) were generally lower in men than for CHD deaths and not statistically significant. The adjusted HRs for non-CHD cardiovascular deaths were higher in women at all levels of daily alcohol intake than for CHD deaths but as for CHD deaths only non-drinkers had a significantly increased risk. There were relatively few cerebral stroke deaths (ICD-10 code I63) in the sample, resulting in wide confidence intervals around the estimated HRs.

# Appendix Table 6. Cox regression results for alcohol consumption and mortality end-points in men free of CVD and cancer at baseline (n=12,357)

|  | **All-cause mortality** | | | | | |  | **CVD mortality (I00-99)** | | | | | |  | **CHD mortality (I20-5)** | | | | | |  | **Alcohol-related mortality** | | | | | |
| --- | --- | --- | --- | --- | --- | --- | --- | --- | --- | --- | --- | --- | --- | --- | --- | --- | --- | --- | --- | --- | --- | --- | --- | --- | --- | --- | --- |
|  | **Age-adjusted Fully-adjusted** | | | | | |  | **Age-adjusted Fully-adjusted** | | | | | |  | **Age-adjusted Fully-adjusted** | | | | | |  | **Age-adjusted Fully-adjusted** | | | | | |
|  | **HR** | **95% CI** | | **HR** | **95% CI** | |  | **HR** | **95% CI** | | **HR** | **95% CI** | |  | **HR** | **95% CI** | | **HR** | **95% CI** | |  | **HR** | **95% CI** | | **HR** | **95% CI** | |
| Alcohol intake |  |  |  |  |  |  |  |  |  |  |  |  |  |  |  |  |  |  |  |  |  |  |  |  |  |  |  |
| Non-drinker | 1.54 | [1.29 | 1.82] | 1.31 | [1.10 | 1.55] |  | 1.53 | [1.17 | 2.01] | 1.29 | [0.98 | 1.69] |  | 1.59 | [1.11 | 2.28] | 1.35 | [0.94 | 1.94] |  | 1.54 | [1.04 | 2.28] | 1.26 | [0.85 | 1.87] |
| <10 g/d | 1.00 | . | . | 1.00 | . | . |  | 1.00 | . | . | 1.00 | . | . |  | 1.00 | . | . | 1.00 | . | . |  | 1.00 | . | . | 1.00 | . | . |
| 10-60 g/d | 1.11 | [0.97 | 1.27] | 1.02 | [0.89 | 1.17] |  | 0.97 | [0.78 | 1.21] | 0.91 | [0.73 | 1.14] |  | 1.06 | [0.79 | 1.42] | 0.97 | [0.72 | 1.31] |  | 1.50 | [1.13 | 1.99] | 1.38 | [1.04 | 1.84] |
| ≥60 g/d | 1.73 | [1.27 | 2.34] | 1.23 | [0.90 | 1.67] |  | 1.91 | [1.20 | 3.03] | 1.33 | [0.83 | 2.13] |  | 2.20 | [1.21 | 4.01] | 1.50 | [0.82 | 2.75] |  | 2.82 | [1.65 | 4.82] | 1.87 | [1.08 | 3.22] |
|  |  |  |  |  |  |  |  |  |  |  |  |  |  |  |  |  |  |  |  |  |  |  |  |  |  |  |  |
| Drinking frequency |  |  |  |  |  |  |  |  |  |  |  |  |  |  |  |  |  |  |  |  |  |  |  |  |  |  |  |
| Never | 1.52 | [1.22 | 1.89] | 1.30 | [1.05 | 1.63] |  | 1.64 | [1.15 | 2.33] | 1.36 | [0.96 | 1.94] |  | 1.48 | [0.94 | 2.32] | 1.24 | [0.79 | 1.95] |  | 1.94 | [1.11 | 3.38] | 1.64 | [0.93 | 2.89] |
| <1 month | 1.00 | . | . | 1.00 | . | . |  | 1.00 | . | . | 1.00 | . | . |  | 1.00 | . | . | 1.00 | . | . |  | 1.00 | . | . | 1.00 | . | . |
| 1-3 month | 1.01 | [0.83 | 1.24] | 1.00 | [0.81 | 1.22] |  | 1.11 | [0.80 | 1.53] | 1.07 | [0.77 | 1.47] |  | 0.96 | [0.64 | 1.46] | 0.92 | [0.60 | 1.39] |  | 1.12 | [0.66 | 1.89] | 1.14 | [0.67 | 1.94] |
| 1-4 week | 1.04 | [0.86 | 1.26] | 1.02 | [0.84 | 1.23] |  | 1.05 | [0.77 | 1.43] | 1.02 | [0.74 | 1.39] |  | 0.91 | [0.61 | 1.35] | 0.87 | [0.58 | 1.29] |  | 1.82 | [1.13 | 2.93] | 1.87 | [1.15 | 3.02] |
| 5+ week | 1.15 | [0.92 | 1.43] | 1.03 | [0.83 | 1.29] |  | 1.16 | [0.81 | 1.66] | 1.04 | [0.72 | 1.49] |  | 1.08 | [0.68 | 1.73] | 0.95 | [0.59 | 1.53] |  | 2.14 | [1.27 | 3.59] | 1.93 | [1.14 | 3.27] |
|  |  |  |  |  |  |  |  |  |  |  |  |  |  |  |  |  |  |  |  |  |  |  |  |  |  |  |  |
| Drinking pattern |  |  |  |  |  |  |  |  |  |  |  |  |  |  |  |  |  |  |  |  |  |  |  |  |  |  |  |
| Non-drinker | 1.56 | [1.28 | 1.90] | 1.26 | [1.03 | 1.54] |  | 1.64 | [1.19 | 2.26] | 1.29 | [0.94 | 1.78] |  | 1.60 | [1.05 | 2.44] | 1.24 | [0.81 | 1.91] |  | 1.36 | [0.87 | 2.13] | 1.06 | [0.68 | 1.67] |
| Light (≤2 drinks/occasion) | 1.00 | . | . | 1.00 | . | . |  | 1.00 | . | . | 1.00 | . | . |  | 1.00 | . | . | 1.00 | . | . |  | 1.00 | . | . | 1.00 | . | . |
| Moderate (≤4 drinks/occasion) | 1.03 | [0.86 | 1.24] | 0.98 | [0.82 | 1.18] |  | 1.05 | [0.78 | 1.42] | 1.01 | [0.75 | 1.35] |  | 1.03 | [0.69 | 1.53] | 0.97 | [0.65 | 1.44] |  | 0.88 | [0.58 | 1.33] | 0.86 | [0.57 | 1.31] |
| Occasional heavy (>4 drinks/occasion <1 wk) | 1.07 | [0.91 | 1.27] | 0.93 | [0.78 | 1.10] |  | 1.08 | [0.82 | 1.42] | 0.92 | [0.70 | 1.22] |  | 1.01 | [0.70 | 1.47] | 0.82 | [0.57 | 1.20] |  | 1.10 | [0.76 | 1.58] | 0.97 | [0.67 | 1.40] |
| Regular heavy (>4 drinks/occasion ≥1 wk) | 1.37 | [1.08 | 1.73] | 1.01 | [0.80 | 1.28] |  | 1.46 | [1.01 | 2.11] | 1.09 | [0.75 | 1.59] |  | 1.50 | [0.93 | 2.42] | 1.07 | [0.66 | 1.73] |  | 1.67 | [1.05 | 2.65] | 1.19 | [0.75 | 1.90] |
|  |  |  |  |  |  |  |  |  |  |  |  |  |  |  |  |  |  |  |  |  |  |  |  |  |  |  |  |
| Binge drinking (100 g ≥1/month) |  |  |  |  |  |  |  |  |  |  |  |  |  |  |  |  |  |  |  |  |  |  |  |  |  |  |  |
| Non-drinker | 1.54 | [1.30 | 1.82] | 1.30 | [1.10 | 1.54] |  | 1.60 | [1.22 | 2.09] | 1.32 | [1.00 | 1.72] |  | 1.59 | [1.12 | 2.27] | 1.31 | [0.92 | 1.87] |  | 1.43 | [0.98 | 2.09] | 1.15 | [0.78 | 1.68] |
| Non-binge drinker | 1.00 | . | . | 1.00 | . | . |  | 1.00 | . | . | 1.00 | . | . |  | 1.00 | . | . | 1.00 | . | . |  | 1.00 | . | . | 1.00 | . | . |
| Binge drinker | 1.25 | [1.08 | 1.44] | 1.01 | [0.88 | 1.17] |  | 1.23 | [0.98 | 1.55] | 0.99 | [0.78 | 1.25] |  | 1.21 | [0.89 | 1.63] | 0.93 | [0.69 | 1.27] |  | 1.51 | [1.14 | 2.02] | 1.19 | [0.89 | 1.60] |
| N | 12357 |  |  | 12357 |  |  |  | 12357 |  |  | 12357 |  |  |  | 12357 |  |  | 12357 |  |  |  | 12357 |  |  | 12357 |  |  |
| No. of cases | 1168 |  |  | 1168 |  |  |  | 451 |  |  | 451 |  |  |  | 253 |  |  | 253 |  |  |  | 254 |  |  | 254 |  |  |
| Person yrs (000s) | 82.43 |  |  | 82.43 |  |  |  | 82.43 |  |  | 82.43 |  |  |  | 82.43 |  |  | 82.43 |  |  |  | 82.43 |  |  | 82.43 |  |  |

Covariates in fully-adjusted model are: age, education, marital status, economic activity, asset score, subjective hardship score, smoking, physical activity, BMI, prevalent CVD and cancer, and depressive symptoms.

# Appendix Table 7. Cox regression results for alcohol consumption and mortality end-points in women free of CVD and cancer at baseline (n=14,233)

|  | **All-cause mortality** | | | | | |  | **CVD mortality (I00-99)** | | | | | |  | **CHD mortality (I20-5)** | | | | | |  | **Alcohol-related mortality** | | | | | |
| --- | --- | --- | --- | --- | --- | --- | --- | --- | --- | --- | --- | --- | --- | --- | --- | --- | --- | --- | --- | --- | --- | --- | --- | --- | --- | --- | --- |
|  | **Age-adjusted Fully-adjusted** | | | | | |  | **Age-adjusted Fully-adjusted** | | | | | |  | **Age-adjusted Fully-adjusted** | | | | | |  | **Age-adjusted Fully-adjusted** | | | | | |
|  | **HR** | **95% CI** | | **HR** | **95% CI** | |  | **HR** | **95% CI** | | **HR** | **95% CI** | |  | **HR** | **95% CI** | | **HR** | **95% CI** | |  | **HR** | **95% CI** | | **HR** | **95% CI** | |
| Alcohol intake |  |  |  |  |  |  |  |  |  |  |  |  |  |  |  |  |  |  |  |  |  |  |  |  |  |  |  |
| Non-drinker | 1.64 | [1.35 | 2.00] | 1.40 | [1.15 | 1.71] |  | 2.16 | [1.57 | 2.97] | 1.79 | [1.31 | 2.46] |  | 1.92 | [1.18 | 3.10] | 1.51 | [0.93 | 2.45] |  | 1.28 | [0.76 | 2.16] | 0.98 | [0.57 | 1.66] |
| <5 g/d | 1.00 | . | . | 1.00 | . | . |  | 1.00 | . | . | 1.00 | . | . |  | 1.00 | . | . | 1.00 | . | . |  | 1.00 | . | . | 1.00 | . | . |
| 5-20 g/d | 0.90 | [0.63 | 1.28] | 0.88 | [0.62 | 1.27] |  | 1.54 | [0.91 | 2.59] | 1.50 | [0.88 | 2.54] |  | 1.01 | [0.40 | 2.57] | 1.02 | [0.40 | 2.60] |  | 0.81 | [0.36 | 1.79] | 0.77 | [0.35 | 1.73] |
| ≥20 g/d | 2.52 | [1.57 | 4.04] | 2.43 | [1.51 | 3.91] |  | 2.57 | [1.04 | 6.37] | 2.44 | [0.97 | 6.13] |  | 2.28 | [0.55 | 9.48] | 2.55 | [0.60 | 10.88] |  | 2.29 | [0.81 | 6.43] | 2.26 | [0.79 | 6.45] |
|  |  |  |  |  |  |  |  |  |  |  |  |  |  |  |  |  |  |  |  |  |  |  |  |  |  |  |  |
| Drinking frequency |  |  |  |  |  |  |  |  |  |  |  |  |  |  |  |  |  |  |  |  |  |  |  |  |  |  |  |
| Never | 1.58 | [1.27 | 1.96] | 1.38 | [1.11 | 1.72] |  | 2.03 | [1.43 | 2.89] | 1.74 | [1.22 | 2.49] |  | 2.04 | [1.19 | 3.52] | 1.69 | [0.98 | 2.94] |  | 1.18 | [0.66 | 2.10] | 0.94 | [0.52 | 1.68] |
| <1 month | 1.00 | . | . | 1.00 | . | . |  | 1.00 | . | . | 1.00 | . | . |  | 1.00 | . | . | 1.00 | . | . |  | 1.00 | . | . | 1.00 | . | . |
| 1-3 month | 0.95 | [0.75 | 1.20] | 0.99 | [0.78 | 1.25] |  | 0.83 | [0.55 | 1.26] | 0.89 | [0.58 | 1.36] |  | 1.09 | [0.61 | 1.95] | 1.23 | [0.69 | 2.20] |  | 0.87 | [0.50 | 1.51] | 0.91 | [0.53 | 1.58] |
| 1-4 week | 0.91 | [0.66 | 1.25] | 0.97 | [0.70 | 1.34] |  | 1.29 | [0.77 | 2.15] | 1.43 | [0.85 | 2.40] |  | 1.33 | [0.59 | 2.99] | 1.54 | [0.68 | 3.50] |  | 0.80 | [0.38 | 1.65] | 0.84 | [0.40 | 1.76] |
| 5+ week | 1.54 | [0.95 | 2.49] | 1.59 | [0.98 | 2.58] |  | 2.06 | [0.93 | 4.59] | 2.06 | [0.91 | 4.64] |  | 1.57 | [0.36 | 6.77] | 1.86 | [0.42 | 8.22] |  | 1.05 | [0.31 | 3.52] | 1.18 | [0.35 | 4.01] |
|  |  |  |  |  |  |  |  |  |  |  |  |  |  |  |  |  |  |  |  |  |  |  |  |  |  |  |  |
| Drinking pattern |  |  |  |  |  |  |  |  |  |  |  |  |  |  |  |  |  |  |  |  |  |  |  |  |  |  |  |
| Non-drinker | 1.52 | [1.16 | 1.99] | 1.30 | [0.99 | 1.70] |  | 1.95 | [1.25 | 3.05] | 1.60 | [1.02 | 2.51] |  | 1.84 | [0.93 | 3.66] | 1.44 | [0.72 | 2.87] |  | 1.43 | [0.69 | 2.97] | 1.11 | [0.53 | 2.31] |
| Light (≤0.5 drink/occasion) | 1.00 | . | . | 1.00 | . | . |  | 1.00 | . | . | 1.00 | . | . |  | 1.00 | . | . | 1.00 | . | . |  | 1.00 | . | . | 1.00 | . | . |
| Moderate (≤ 2 drinks/occasion) | 0.85 | [0.65 | 1.12] | 0.87 | [0.66 | 1.13] |  | 0.83 | [0.52 | 1.32] | 0.83 | [0.52 | 1.32] |  | 0.95 | [0.49 | 1.87] | 0.96 | [0.49 | 1.89] |  | 1.13 | [0.56 | 2.25] | 1.17 | [0.59 | 2.34] |
| Occasional heavy (>2 drinks/occasion <1 week) | 1.07 | [0.79 | 1.45] | 0.98 | [0.72 | 1.33] |  | 1.33 | [0.79 | 2.25] | 1.14 | [0.67 | 1.93] |  | 1.17 | [0.53 | 2.58] | 1.00 | [0.45 | 2.22] |  | 1.09 | [0.50 | 2.37] | 1.01 | [0.46 | 2.21] |
| Regular heavy (>2 drinks/occasion ≥1 week) | 1.79 | [0.89 | 3.58] | 1.42 | [0.70 | 2.86] |  | 0.84 | [0.11 | 6.13] | 0.61 | [0.08 | 4.64] |  | NA |  |  | NA |  |  |  | 2.83 | [0.77 | 10.33] | 2.34 | [0.63 | 8.66] |
|  |  |  |  |  |  |  |  |  |  |  |  |  |  |  |  |  |  |  |  |  |  |  |  |  |  |  |  |
| Binge drinking (60 g ≥1/month) |  |  |  |  |  |  |  |  |  |  |  |  |  |  |  |  |  |  |  |  |  |  |  |  |  |  |  |
| Non-drinker | 1.65 | [1.36 | 2.01] | 1.42 | [1.16 | 1.72] |  | 2.15 | [1.57 | 2.95] | 1.79 | [1.31 | 2.46] |  | 1.98 | [1.22 | 3.19] | 1.56 | [0.96 | 2.53] |  | 1.31 | [0.77 | 2.20] | 1.00 | [0.59 | 1.69] |
| Non-binge drinker | 1.00 | . | . | 1.00 | . | . |  | 1.00 | . | . | 1.00 | . | . |  | 1.00 | . | . | 1.00 | . | . |  | 1.00 | . | . | 1.00 | . | . |
| Binge drinker | 1.35 | [0.94 | 1.93] | 1.17 | [0.81 | 1.69] |  | 2.16 | [1.25 | 3.74] | 1.84 | [1.05 | 3.21] |  | 1.97 | [0.84 | 4.66] | 1.82 | [0.76 | 4.39] |  | 1.38 | [0.65 | 2.93] | 1.15 | [0.54 | 2.46] |
| N | 14233 |  |  | 14233 |  |  |  | 14233 |  |  | 14233 |  |  |  | 14233 |  |  | 14233 |  |  |  | 14233 |  |  | 14233 |  |  |
| No. of cases | 570 |  |  | 570 |  |  |  | 210 |  |  | 210 |  |  |  | 92 |  |  | 92 |  |  |  | 90 |  |  | 90 |  |  |
| Person yrs (000s) | 98.1 |  |  | 98.1 |  |  |  | 98.1 |  |  | 98.1 |  |  |  | 98.1 |  |  | 98.1 |  |  |  | 98.1 |  |  | 98.1 |  |  |

Covariates in fully-adjusted model are: age, education, marital status, economic activity, asset score, subjective hardship score, smoking, physical activity, BMI, prevalent CVD and cancer, and depressive symptoms.

# Appendix Table 8. Cox regression results for alcohol consumption and mortality end-points in Novosibirsk & Kaunas vs. Czech & Krakow men

|  | **All-cause mortality** | | | | | |  | **CVD (I00-99) mortality** | | | | | |  | **CHD (I20-5) mortality** | | | | | |  | **Alcohol-related mortality** | | | | | |
| --- | --- | --- | --- | --- | --- | --- | --- | --- | --- | --- | --- | --- | --- | --- | --- | --- | --- | --- | --- | --- | --- | --- | --- | --- | --- | --- | --- |
|  | **Age-adjusted** | | | **Fully-adjusted** | | |  | **Age-adjusted** | | | **Fully-adjusted** | | |  | **Age-adjusted** | | | **Fully-adjusted** | | |  | **Age-adjusted** | | | **Fully-adjusted** | | |
|  | **HR** | **95% CI** | | **HR** | **95% CI** | |  | **HR** | **95% CI** | | **HR** | **95% CI** | |  | **HR** | **95% CI** | | **HR** | **95% CI** | |  | **HR** | **95% CI** | | **HR** | **95% CI** | |
| **Novosibirsk & Kaunas** |  |  |  |  |  |  |  |  |  |  |  |  |  |  |  |  |  |  |  |  |  |  |  |  |  |  |  |
| Alcohol intake |  |  |  |  |  |  |  |  |  |  |  |  |  |  |  |  |  |  |  |  |  |  |  |  |  |  |  |
| Non-drinker | 1.43 | [1.18 | 1.73] | 1.18 | [0.97 | 1.43] |  | 1.34 | [1.03 | 1.75] | 1.11 | [0.85 | 1.45] |  | 1.49 | [1.08 | 2.06] | 1.24 | [0.89 | 1.72] |  | 1.72 | [1.10 | 2.71] | 1.40 | [0.89 | 2.21] |
| <10 g/d | 1.00 | . | . | 1.00 | . | . |  | 1.00 | . | . | 1.00 | . | . |  | 1.00 | . | . | 1.00 | . | . |  | 1.00 | . | . | 1.00 | . | . |
| 10-60 g/d | 0.96 | [0.83 | 1.11] | 0.96 | [0.82 | 1.12] |  | 0.79 | [0.64 | 0.99] | 0.83 | [0.66 | 1.04] |  | 0.90 | [0.69 | 1.18] | 0.93 | [0.71 | 1.23] |  | 1.51 | [1.09 | 2.11] | 1.45 | [1.03 | 2.04] |
| ≥60 g/d | 1.86 | [1.28 | 2.69] | 1.47 | [1.01 | 2.14] |  | 1.74 | [1.01 | 2.99] | 1.44 | [0.83 | 2.48] |  | 1.81 | [0.92 | 3.55] | 1.56 | [0.79 | 3.07] |  | 4.42 | [2.44 | 8.01] | 3.17 | [1.73 | 5.81] |
|  |  |  |  |  |  |  |  |  |  |  |  |  |  |  |  |  |  |  |  |  |  |  |  |  |  |  |  |
| Drinking frequency |  |  |  |  |  |  |  |  |  |  |  |  |  |  |  |  |  |  |  |  |  |  |  |  |  |  |  |
| Never | 1.39 | [1.10 | 1.76] | 1.20 | [0.95 | 1.51] |  | 1.30 | [0.94 | 1.80] | 1.13 | [0.82 | 1.56] |  | 1.33 | [0.90 | 1.96] | 1.15 | [0.78 | 1.71] |  | 1.87 | [1.02 | 3.42] | 1.59 | [0.87 | 2.93] |
| <1 month | 1.00 | . | . | 1.00 | . | . |  | 1.00 | . | . | 1.00 | . | . |  | 1.00 | . | . | 1.00 | . | . |  | 1.00 | . | . | 1.00 | . | . |
| 1-3 month | 0.99 | [0.81 | 1.20] | 1.02 | [0.84 | 1.25] |  | 0.98 | [0.74 | 1.28] | 1.03 | [0.78 | 1.35] |  | 0.87 | [0.62 | 1.22] | 0.91 | [0.65 | 1.29] |  | 1.03 | [0.58 | 1.81] | 1.06 | [0.60 | 1.88] |
| 1-4 week | 0.93 | [0.76 | 1.13] | 0.99 | [0.81 | 1.21] |  | 0.80 | [0.60 | 1.05] | 0.88 | [0.67 | 1.17] |  | 0.79 | [0.56 | 1.11] | 0.87 | [0.62 | 1.22] |  | 1.55 | [0.92 | 2.60] | 1.63 | [0.97 | 2.76] |
| 5+ week | 1.09 | [0.83 | 1.42] | 1.07 | [0.82 | 1.40] |  | 1.02 | [0.70 | 1.49] | 1.08 | [0.74 | 1.58] |  | 0.97 | [0.60 | 1.54] | 1.03 | [0.64 | 1.66] |  | 2.40 | [1.32 | 4.36] | 2.21 | [1.20 | 4.05] |
|  |  |  |  |  |  |  |  |  |  |  |  |  |  |  |  |  |  |  |  |  |  |  |  |  |  |  |  |
| Drinking pattern |  |  |  |  |  |  |  |  |  |  |  |  |  |  |  |  |  |  |  |  |  |  |  |  |  |  |  |
| Non-drinker | 1.48 | [1.17 | 1.87] | 1.23 | [0.97 | 1.56] |  | 1.44 | [1.04 | 1.99] | 1.22 | [0.88 | 1.70] |  | 1.54 | [1.03 | 2.29] | 1.33 | [0.89 | 2.00] |  | 1.75 | [0.98 | 3.14] | 1.40 | [0.78 | 2.52] |
| Light (QPO ≤40 g) | 1.00 | . | . | 1.00 | . | . |  | 1.00 | . | . | 1.00 | . | . |  | 1.00 | . | . | 1.00 | . | . |  | 1.00 | . | . | 1.00 | . | . |
| Moderate (QPO ≤80 g) | 1.05 | [0.86 | 1.29] | 1.11 | [0.91 | 1.36] |  | 1.04 | [0.78 | 1.38] | 1.15 | [0.87 | 1.53] |  | 1.07 | [0.75 | 1.53] | 1.21 | [0.85 | 1.73] |  | 1.15 | [0.68 | 1.97] | 1.22 | [0.71 | 2.10] |
| Occasional heavy (QPO >80 g <1 pw) | 0.99 | [0.82 | 1.20] | 1.00 | [0.82 | 1.21] |  | 1.00 | [0.76 | 1.31] | 1.05 | [0.79 | 1.38] |  | 0.99 | [0.70 | 1.39] | 1.05 | [0.74 | 1.49] |  | 1.23 | [0.75 | 2.02] | 1.16 | [0.70 | 1.93] |
| Regular heavy (QPO >80 g ≥1 pw) | 1.14 | [0.88 | 1.47] | 1.05 | [0.81 | 1.37] |  | 0.96 | [0.66 | 1.40] | 0.97 | [0.66 | 1.43] |  | 0.97 | [0.60 | 1.56] | 0.99 | [0.61 | 1.60] |  | 2.10 | [1.20 | 3.69] | 1.69 | [0.95 | 3.02] |
|  |  |  |  |  |  |  |  |  |  |  |  |  |  |  |  |  |  |  |  |  |  |  |  |  |  |  |  |
| Binge drinking (100 g ≥1/month) |  |  |  |  |  |  |  |  |  |  |  |  |  |  |  |  |  |  |  |  |  |  |  |  |  |  |  |
| Non-drinker | 1.46 | [1.21 | 1.77] | 1.18 | [0.97 | 1.43] |  | 1.43 | [1.09 | 1.86] | 1.14 | [0.87 | 1.49] |  | 1.48 | [1.07 | 2.04] | 1.19 | [0.86 | 1.64] |  | 1.62 | [1.04 | 2.51] | 1.26 | [0.80 | 1.97] |
| Non-binge drinker | 1.00 | . | . | 1.00 | . | . |  | 1.00 | . | . | 1.00 | . | . |  | 1.00 | . | . | 1.00 | . | . |  | 1.00 | . | . | 1.00 | . | . |
| Binge drinker | 1.09 | [0.94 | 1.27] | 1.00 | [0.86 | 1.16] |  | 1.01 | [0.81 | 1.26] | 0.95 | [0.76 | 1.19] |  | 0.91 | [0.69 | 1.20] | 0.86 | [0.65 | 1.14] |  | 1.59 | [1.15 | 2.18] | 1.30 | [0.94 | 1.81] |
| N/No. of deaths/ Person yrs (000s) | 7371 | 975 | 42.1 | 7371 | 975 | 42.1 |  | 7371 | 479 | 42.1 | 7371 | 479 | 42.1 |  | 7371 | 308 | 42.1 | 7371 | 308 | 42.1 |  | 7371 | 185 | 42.1 | 7371 | 185 | 42.1 |
|  |  |  |  |  |  |  |  |  |  |  |  |  |  |  |  |  |  |  |  |  |  |  |  |  |  |  |  |
| **Czech towns & Krakow** |  |  |  |  |  |  |  |  |  |  |  |  |  |  |  |  |  |  |  |  |  |  |  |  |  |  |  |
| Alcohol intake |  |  |  |  |  |  |  |  |  |  |  |  |  |  |  |  |  |  |  |  |  |  |  |  |  |  |  |
| Non-drinker | 1.77 | [1.50 | 2.08] | 1.51 | [1.28 | 1.78] |  | 1.92 | [1.47 | 2.49] | 1.59 | [1.22 | 1.92] |  | 2.02 | [1.42 | 2.89] | 1.73 | [1.20 | 2.47] |  | 1.62 | [1.09 | 2.41] | 1.30 | [0.87 | 1.94] |
| <10 g/d | 1.00 | . | . | 1.00 | . | . |  | 1.00 | . | . | 1.00 | . | 1.00 |  | 1.00 | . | . | 1.00 | . | . |  | 1.00 | . | . | 1.00 | . | . |
| 10-60 g/d | 1.10 | [0.93 | 1.29] | 1.04 | [0.88 | 1.22] |  | 0.93 | [0.70 | 1.23] | 0.90 | [0.68 | 0.93] |  | 0.92 | [0.62 | 1.37] | 0.88 | [0.59 | 1.32] |  | 1.26 | [0.88 | 1.80] | 1.20 | [0.84 | 1.73] |
| ≥60 g/d | 1.44 | [1.01 | 2.05] | 1.07 | [0.75 | 1.53] |  | 1.73 | [1.03 | 2.90] | 1.36 | [0.80 | 1.73] |  | 2.26 | [1.16 | 4.40] | 1.71 | [0.87 | 3.37] |  | 1.92 | [0.95 | 3.87] | 1.34 | [0.66 | 2.74] |
|  |  |  |  |  |  |  |  |  |  |  |  |  |  |  |  |  |  |  |  |  |  |  |  |  |  |  |  |
| Drinking frequency |  |  |  |  |  |  |  |  |  |  |  |  |  |  |  |  |  |  |  |  |  |  |  |  |  |  |  |
| Never | 1.69 | [1.36 | 2.10] | 1.43 | [1.15 | 1.78] |  | 1.79 | [1.26 | 2.54] | 1.48 | [1.04 | 1.79] |  | 1.91 | [1.18 | 3.09] | 1.61 | [0.99 | 2.61] |  | 2.29 | [1.25 | 4.20] | 1.86 | [1.01 | 3.41] |
| <1 month | 1.00 | . | . | 1.00 | . | . |  | 1.00 | . | . | 1.00 | . | 1.00 |  | 1.00 | . | . | 1.00 | . | . |  | 1.00 | . | . | 1.00 | . | . |
| 1-3 month | 0.98 | [0.78 | 1.23] | 0.94 | [0.75 | 1.18] |  | 0.99 | [0.69 | 1.42] | 0.95 | [0.66 | 0.99] |  | 1.10 | [0.67 | 1.82] | 1.04 | [0.63 | 1.71] |  | 1.59 | [0.87 | 2.88] | 1.57 | [0.86 | 2.84] |
| 1-4 week | 0.96 | [0.77 | 1.19] | 0.95 | [0.77 | 1.18] |  | 0.82 | [0.57 | 1.16] | 0.82 | [0.58 | 0.82] |  | 0.70 | [0.42 | 1.17] | 0.69 | [0.41 | 1.16] |  | 1.62 | [0.92 | 2.86] | 1.67 | [0.94 | 2.95] |
| 5+ week | 1.04 | [0.83 | 1.31] | 0.99 | [0.79 | 1.25] |  | 0.97 | [0.67 | 1.41] | 0.97 | [0.67 | 0.97] |  | 1.14 | [0.68 | 1.90] | 1.11 | [0.66 | 1.87] |  | 1.87 | [1.03 | 3.39] | 1.79 | [0.99 | 3.26] |
|  |  |  |  |  |  |  |  |  |  |  |  |  |  |  |  |  |  |  |  |  |  |  |  |  |  |  |  |
| Drinking pattern |  |  |  |  |  |  |  |  |  |  |  |  |  |  |  |  |  |  |  |  |  |  |  |  |  |  |  |
| Non-drinker | 1.76 | [1.47 | 2.10] | 1.49 | [1.24 | 1.78] |  | 1.93 | [1.44 | 2.57] | 1.60 | [1.20 | 1.93] |  | 2.09 | [1.41 | 3.11] | 1.78 | [1.20 | 2.65] |  | 1.55 | [1.01 | 2.39] | 1.24 | [0.80 | 1.92] |
| Light (QPO ≤40 g) | 1.00 | . | . | 1.00 | . | . |  | 1.00 | . | . | 1.00 | . | 1.00 |  | 1.00 | . | . | 1.00 | . | . |  | 1.00 | . | . | 1.00 | . | . |
| Moderate (QPO ≤80 g) | 1.01 | [0.84 | 1.23] | 0.99 | [0.81 | 1.20] |  | 0.98 | [0.72 | 1.35] | 0.97 | [0.71 | 0.98] |  | 0.93 | [0.59 | 1.47] | 0.92 | [0.58 | 1.45] |  | 0.96 | [0.61 | 1.50] | 0.95 | [0.61 | 1.49] |
| Occasional heavy (QPO >80 g <1 pw) | 1.05 | [0.88 | 1.26] | 1.02 | [0.85 | 1.22] |  | 1.00 | [0.74 | 1.36] | 1.02 | [0.75 | 1.00] |  | 1.15 | [0.76 | 1.74] | 1.13 | [0.75 | 1.72] |  | 1.11 | [0.74 | 1.65] | 1.10 | [0.74 | 1.65] |
| Regular heavy (QPO >80 g ≥1 pw) | 1.24 | [0.90 | 1.71] | 0.96 | [0.69 | 1.32] |  | 1.42 | [0.86 | 2.33] | 1.13 | [0.68 | 1.42] |  | 1.73 | [0.89 | 3.36] | 1.34 | [0.68 | 2.61] |  | 1.28 | [0.64 | 2.55] | 0.95 | [0.47 | 1.92] |
|  |  |  |  |  |  |  |  |  |  |  |  |  |  |  |  |  |  |  |  |  |  |  |  |  |  |  |  |
| Binge drinking (100 g ≥1/month) |  |  |  |  |  |  |  |  |  |  |  |  |  |  |  |  |  |  |  |  |  |  |  |  |  |  |  |
| Non-drinker | 1.77 | [1.51 | 2.07] | 1.50 | [1.28 | 1.76] |  | 1.97 | [1.52 | 2.55] | 1.63 | [1.26 | 1.97] |  | 2.15 | [1.51 | 3.05] | 1.83 | [1.28 | 2.60] |  | 1.57 | [1.07 | 2.31] | 1.25 | [0.85 | 1.85] |
| Non-binge drinker | 1.00 | . | . | 1.00 | . | . |  | 1.00 | . | . | 1.00 | . | 1.00 |  | 1.00 | . | . | 1.00 | . | . |  | 1.00 | . | . | 1.00 | . | . |
| Binge drinker | 1.24 | [1.02 | 1.51] | 1.04 | [0.86 | 1.27] |  | 1.22 | [0.88 | 1.70] | 1.08 | [0.78 | 1.22] |  | 1.63 | [1.07 | 2.47] | 1.38 | [0.90 | 2.10] |  | 1.39 | [0.93 | 2.09] | 1.16 | [0.77 | 1.76] |
| N/No. of deaths/ Person yrs (000s) | 8618 | 964 | 63.1 | 8618 | 964 | 63.1 |  | 8618 | 354 | 63.1 | 8618 | 354 | 8618 |  | 8618 | 185 | 63.1 | 8618 | 185 | 63.1 |  | 8618 | 184 | 63.1 | 8618 | 184 | 63.1 |

# Appendix Table 9. Cox regression results for alcohol consumption and mortality end-points in Novosibirsk & Kaunas vs. Czech & Krakow women

|  | **All-cause mortality** | | | | | |  | **CVD (I00-99) mortality** | | | | | |  | **CHD (I20-5) mortality** | | | | | |  | **Alcohol-related mortality** | | | | | |
| --- | --- | --- | --- | --- | --- | --- | --- | --- | --- | --- | --- | --- | --- | --- | --- | --- | --- | --- | --- | --- | --- | --- | --- | --- | --- | --- | --- |
|  | **Age-adjusted** | | | **Fully-adjusted** | | |  | **Age-adjusted** | | | **Fully-adjusted** | | |  | **Age-adjusted** | | | **Fully-adjusted** | | |  | **Age-adjusted** | | | **Fully-adjusted** | | |
|  | **HR** | **95% CI** | | **HR** | **95% CI** | |  | **HR** | **95% CI** | | **HR** | **95% CI** | |  | **HR** | **95% CI** | | **HR** | **95% CI** | |  | **HR** | **95% CI** | | **HR** | **95% CI** | |
| **Novosibirsk & Kaunas** |  |  |  |  |  |  |  |  |  |  |  |  |  |  |  |  |  |  |  |  |  |  |  |  |  |  |  |
| Alcohol intake |  |  |  |  |  |  |  |  |  |  |  |  |  |  |  |  |  |  |  |  |  |  |  |  |  |  |  |
| Non-drinker | 1.58 | [1.25 | 1.99] | 1.33 | [1.05 | 1.68] |  | 1.78 | [1.31 | 2.43] | 1.39 | [1.01 | 1.91] |  | 1.74 | [1.15 | 2.65] | 1.32 | [0.86 | 2.02] |  | 1.64 | [0.82 | 3.28] | 1.50 | [0.74 | 3.05] |
| <5 g/d | 1.00 | . | . | 1.00 | . | . |  | 1.00 | . | . | 1.00 | . | . |  | 1.00 | . | . | 1.00 | . | . |  | 1.00 | . | . | 1.00 | . | . |
| 5-20 g/d | 1.13 | [0.74 | 1.73] | 1.09 | [0.72 | 1.67] |  | 1.17 | [0.59 | 2.32] | 1.05 | [0.53 | 2.09] |  | 0.72 | [0.23 | 2.30] | 0.67 | [0.21 | 2.17] |  | 1.88 | [0.78 | 4.55] | 1.59 | [0.65 | 3.89] |
| ≥20 g/d | 1.52 | [0.57 | 4.09] | 1.31 | [0.48 | 3.57] |  | 0.99 | [0.14 | 7.11] | 0.86 | [0.12 | 6.33] |  | 1.78 | [0.25 | 12.79] | 1.79 | [0.24 | 13.41] |  |  |  |  |  |  |  |
|  |  |  |  |  |  |  |  |  |  |  |  |  |  |  |  |  |  |  |  |  |  |  |  |  |  |  |  |
| Drinking frequency |  |  |  |  |  |  |  |  |  |  |  |  |  |  |  |  |  |  |  |  |  |  |  |  |  |  |  |
| Never | 1.54 | [1.21 | 1.97] | 1.34 | [1.04 | 1.72] |  | 1.59 | [1.15 | 2.19] | 1.29 | [0.92 | 1.80] |  | 1.61 | [1.04 | 2.50] | 1.27 | [0.81 | 1.99] |  | 1.47 | [0.71 | 3.06] | 1.37 | [0.65 | 2.87] |
| <1 month | 1.00 | . | . | 1.00 | . | . |  | 1.00 | . | . | 1.00 | . | . |  | 1.00 | . | . | 1.00 | . | . |  | 1.00 | . | . | 1.00 | . | . |
| 1-3 month | 0.96 | [0.76 | 1.23] | 1.05 | [0.82 | 1.34] |  | 0.70 | [0.48 | 1.03] | 0.79 | [0.53 | 1.16] |  | 0.77 | [0.47 | 1.28] | 0.88 | [0.53 | 1.46] |  | 0.84 | [0.43 | 1.67] | 0.84 | [0.42 | 1.68] |
| 1-4 week | 0.83 | [0.53 | 1.30] | 0.88 | [0.56 | 1.39] |  | 0.69 | [0.33 | 1.46] | 0.74 | [0.35 | 1.58] |  | 0.69 | [0.24 | 1.99] | 0.77 | [0.26 | 2.28] |  | 0.86 | [0.29 | 2.54] | 0.77 | [0.26 | 2.31] |
| 5+ week | 2.03 | [0.95 | 4.36] | 2.15 | [1.00 | 4.65] |  | 0.66 | [0.09 | 4.81] | 0.73 | [0.10 | 5.34] |  |  |  |  |  |  |  |  | 2.19 | [0.29 | 16.50] | 1.92 | [0.25 | 14.65] |
|  |  |  |  |  |  |  |  |  |  |  |  |  |  |  |  |  |  |  |  |  |  |  |  |  |  |  |  |
| Drinking pattern |  |  |  |  |  |  |  |  |  |  |  |  |  |  |  |  |  |  |  |  |  |  |  |  |  |  |  |
| Non-drinker | 1.54 | [1.11 | 2.14] | 1.30 | [0.94 | 1.82] |  | 1.93 | [1.20 | 3.11] | 1.55 | [0.96 | 2.50] |  | 1.95 | [1.03 | 3.70] | 1.54 | [0.81 | 2.95] |  | 1.88 | [0.65 | 5.44] | 1.53 | [0.53 | 4.47] |
| Light (QPO ≤10 g) | 1.00 | . | . | 1.00 | . | . |  | 1.00 | . | . | 1.00 | . | . |  | 1.00 | . | . | 1.00 | . | . |  | 1.00 | . | . | 1.00 | . | . |
| Moderate (QPO ≤40 g) | 0.97 | [0.72 | 1.31] | 1.00 | [0.74 | 1.35] |  | 1.09 | [0.69 | 1.71] | 1.16 | [0.74 | 1.83] |  | 1.19 | [0.65 | 2.17] | 1.28 | [0.70 | 2.36] |  | 1.28 | [0.49 | 3.35] | 1.17 | [0.45 | 3.08] |
| Occasional heavy (QPO >40 g <1 pw) | 1.02 | [0.71 | 1.46] | 0.92 | [0.64 | 1.33] |  | 1.25 | [0.72 | 2.16] | 1.08 | [0.62 | 1.90] |  | 1.05 | [0.48 | 2.29] | 0.96 | [0.43 | 2.12] |  | 1.05 | [0.34 | 3.20] | 0.73 | [0.23 | 2.29] |
| Regular heavy (QPO >40 g ≥1 pw) | 1.45 | [0.52 | 4.01] | 1.11 | [0.40 | 3.11] |  | 1.05 | [0.14 | 7.80] | 0.69 | [0.09 | 5.28] |  | 0.00 | [0.00 | 0.00] | 0.00 | [0.00 | 0.00] |  | 2.37 | [0.27 | 20.63] | 1.41 | [0.16 | 12.74] |
|  |  |  |  |  |  |  |  |  |  |  |  |  |  |  |  |  |  |  |  |  |  |  |  |  |  |  |  |
| Binge drinking (60 g ≥1/month) |  |  |  |  |  |  |  |  |  |  |  |  |  |  |  |  |  |  |  |  |  |  |  |  |  |  |  |
| Non-drinker | 1.57 | [1.25 | 1.98] | 1.32 | [1.04 | 1.67] |  | 1.80 | [1.32 | 2.46] | 1.40 | [1.02 | 1.93] |  | 1.79 | [1.18 | 2.72] | 1.35 | [0.88 | 2.08] |  | 1.59 | [0.80 | 3.18] | 1.47 | [0.73 | 2.97] |
| Non-binge drinker | 1.00 | . | . | 1.00 | . | . |  | 1.00 | . | . | 1.00 | . | . |  | 1.00 | . | . | 1.00 | . | . |  | 1.00 | . | . | 1.00 | . | . |
| Binge drinker | 1.08 | [0.67 | 1.72] | 0.93 | [0.58 | 1.51] |  | 1.47 | [0.74 | 2.92] | 1.10 | [0.54 | 2.24] |  | 1.52 | [0.61 | 3.80] | 1.25 | [0.48 | 3.22] |  | 1.31 | [0.46 | 3.75] | 1.03 | [0.35 | 2.99] |
| N/No. of deaths/ Person yrs (000s) | 8910 | 431 | 54.0 | 8910 | 431 | 54.0 |  | 8910 | 207 | 54.0 | 8910 | 207 | 54.0 |  | 8910 | 116 | 54.0 | 8910 | 116 | 54.0 |  | 8910 | 52 | 54.0 | 8910 | 52 | 54.0 |
|  |  |  |  |  |  |  |  |  |  |  |  |  |  |  |  |  |  |  |  |  |  |  |  |  |  |  |  |
| **Czech towns & Krakow** |  |  |  |  |  |  |  |  |  |  |  |  |  |  |  |  |  |  |  |  |  |  |  |  |  |  |  |
| Alcohol intake |  |  |  |  |  |  |  |  |  |  |  |  |  |  |  |  |  |  |  |  |  |  |  |  |  |  |  |
| Non-drinker | 1.67 | [1.38 | 2.03] | 1.38 | [1.14 | 1.68] |  | 2.31 | [1.64 | 3.24] | 1.86 | [1.32 | 2.62] |  | 2.14 | [1.21 | 3.76] | 1.57 | [0.89 | 2.78] |  | 1.10 | [0.63 | 1.93] | 0.79 | [0.45 | 1.39] |
| <5 g/d | 1.00 | . | . | 1.00 | . | . |  | 1.00 | . | . | 1.00 | . | . |  | 1.00 | . | . | 1.00 | . | . |  | 1.00 | . | . | 1.00 | . | . |
| 5-20 g/d | 0.76 | [0.50 | 1.14] | 0.78 | [0.52 | 1.18] |  | 1.31 | [0.71 | 2.43] | 1.35 | [0.73 | 2.51] |  | 1.10 | [0.37 | 3.22] | 1.25 | [0.42 | 3.68] |  | 0.57 | [0.20 | 1.61] | 0.63 | [0.22 | 1.80] |
| ≥20 g/d | 2.18 | [1.35 | 3.50] | 2.09 | [1.30 | 3.37] |  | 2.35 | [0.94 | 5.90] | 2.24 | [0.88 | 5.66] |  | 1.26 | [0.17 | 9.38] | 1.21 | [0.16 | 9.15] |  | 3.57 | [1.48 | 8.64] | 4.05 | [1.64 | 9.98] |
|  |  |  |  |  |  |  |  |  |  |  |  |  |  |  |  |  |  |  |  |  |  |  |  |  |  |  |  |
| Drinking frequency |  |  |  |  |  |  |  |  |  |  |  |  |  |  |  |  |  |  |  |  |  |  |  |  |  |  |  |
| Never | 1.60 | [1.27 | 2.01] | 1.36 | [1.08 | 1.71] |  | 2.22 | [1.46 | 3.37] | 1.83 | [1.21 | 2.78] |  | 2.51 | [1.19 | 5.32] | 1.91 | [0.90 | 4.05] |  | 1.21 | [0.62 | 2.36] | 0.92 | [0.47 | 1.80] |
| <1 month | 1.00 | . | . | 1.00 | . | . |  | 1.00 | . | . | 1.00 | . | . |  | 1.00 | . | . | 1.00 | . | . |  | 1.00 | . | . | 1.00 | . | . |
| 1-3 month | 0.90 | [0.68 | 1.20] | 0.93 | [0.70 | 1.25] |  | 0.78 | [0.44 | 1.41] | 0.80 | [0.44 | 1.44] |  | 1.25 | [0.49 | 3.16] | 1.30 | [0.51 | 3.33] |  | 1.13 | [0.55 | 2.33] | 1.21 | [0.59 | 2.49] |
| 1-4 week | 0.89 | [0.64 | 1.24] | 1.00 | [0.71 | 1.40] |  | 1.31 | [0.74 | 2.34] | 1.52 | [0.85 | 2.72] |  | 1.35 | [0.48 | 3.79] | 1.68 | [0.59 | 4.80] |  | 1.22 | [0.56 | 2.67] | 1.52 | [0.69 | 3.38] |
| 5+ week | 1.14 | [0.70 | 1.88] | 1.16 | [0.71 | 1.92] |  | 1.93 | [0.88 | 4.22] | 1.85 | [0.84 | 4.08] |  | 1.60 | [0.35 | 7.44] | 1.69 | [0.36 | 7.95] |  | 1.17 | [0.33 | 4.07] | 1.64 | [0.46 | 5.80] |
|  |  |  |  |  |  |  |  |  |  |  |  |  |  |  |  |  |  |  |  |  |  |  |  |  |  |  |  |
| Drinking pattern |  |  |  |  |  |  |  |  |  |  |  |  |  |  |  |  |  |  |  |  |  |  |  |  |  |  |  |
| Non-drinker | 1.51 | [1.17 | 1.95] | 1.28 | [0.99 | 1.66] |  | 1.69 | [1.11 | 2.57] | 1.41 | [0.92 | 2.16] |  | 1.43 | [0.74 | 2.78] | 1.13 | [0.58 | 2.21] |  | 1.53 | [0.68 | 3.44] | 1.14 | [0.51 | 2.58] |
| Light (QPO ≤10 g) | 1.00 | . | . | 1.00 | . | . |  | 1.00 | . | . | 1.00 | . | . |  | 1.00 | . | . | 1.00 | . | . |  | 1.00 | . | . | 1.00 | . | . |
| Moderate (QPO ≤40 g) | 0.76 | [0.56 | 1.03] | 0.82 | [0.60 | 1.11] |  | 0.63 | [0.37 | 1.07] | 0.69 | [0.40 | 1.18] |  | 0.46 | [0.19 | 1.12] | 0.54 | [0.22 | 1.30] |  | 1.15 | [0.48 | 2.75] | 1.26 | [0.53 | 3.01] |
| Occasional heavy (QPO >40 g <1 pw) | 0.96 | [0.68 | 1.35] | 0.95 | [0.68 | 1.34] |  | 0.77 | [0.41 | 1.44] | 0.75 | [0.40 | 1.42] |  | 0.58 | [0.20 | 1.68] | 0.62 | [0.21 | 1.84] |  | 2.05 | [0.85 | 4.92] | 2.15 | [0.89 | 5.17] |
| Regular heavy (QPO >40 g ≥1 pw) | 1.82 | [0.91 | 3.64] | 1.50 | [0.75 | 3.01] |  |  |  |  |  |  |  |  |  |  |  |  |  |  |  | 8.45 | [2.67 | 26.71] | 7.31 | [2.28 | 23.42] |
|  |  |  |  |  |  |  |  |  |  |  |  |  |  |  |  |  |  |  |  |  |  |  |  |  |  |  |  |
| Binge drinking (60 g ≥1/month) |  |  |  |  |  |  |  |  |  |  |  |  |  |  |  |  |  |  |  |  |  |  |  |  |  |  |  |
| Non-drinker | 1.74 | [1.44 | 2.11] | 1.42 | [1.17 | 1.73] |  | 2.30 | [1.65 | 3.22] | 1.84 | [1.31 | 2.59] |  | 2.16 | [1.24 | 3.77] | 1.56 | [0.89 | 2.74] |  | 1.16 | [0.67 | 2.01] | 0.81 | [0.46 | 1.42] |
| Non-binge drinker | 1.00 | . | . | 1.00 | . | . |  | 1.00 | . | . | 1.00 | . | . |  | 1.00 | . | . | 1.00 | . | . |  | 1.00 | . | . | 1.00 | . | . |
| Binge drinker | 1.43 | [0.95 | 2.15] | 1.24 | [0.82 | 1.86] |  | 1.91 | [0.95 | 3.86] | 1.66 | [0.82 | 3.36] |  | 1.21 | [0.28 | 5.16] | 1.13 | [0.26 | 4.85] |  | 1.93 | [0.85 | 4.39] | 1.60 | [0.69 | 3.70] |
| N/No. of deaths/ Person yrs (000s) | 9405 | 525 | 71.0 | 9405 | 525 | 71.0 |  | 9405 | 182 | 71.0 | 9405 | 182 | 71.0 |  | 9405 | 63 | 71.0 | 9405 | 63 | 71.0 |  | 9405 | 68 | 71.0 | 9405 | 68 | 71.0 |

**Past drinking behavior and mortality in Novosibirsk**

**Methods**

At baseline, participants in Novosibirsk were asked whether they used to drink more in the past, compared to how much they were drinking now. This information was combined with reports about participants’ current alcohol consumption, to distinguish between the following categories of drinkers: stable non-drinker (non-drinker at baseline, reported not drinking more in the past), stable drinker (drank alcohol at baseline, did not drink more in the past), cut down (drank alcohol at baseline, reported drinking more in the past), and ex-drinker (non-drinker at baseline, reported drinking more in the past).

# Appendix Table 10. Cox regression results for past drinking behavior (before baseline) and mortality end-points in Novosibirsk

|  |  |  |  |  |  | **All-cause**  **mortality** | | | | | |  | **CVD (I00-99)**  **mortality** | | | | | |  | **CHD (I20-5)**  **mortality** | | | | | |  | **Alcohol-related**  **mortality** | | | | | |
| --- | --- | --- | --- | --- | --- | --- | --- | --- | --- | --- | --- | --- | --- | --- | --- | --- | --- | --- | --- | --- | --- | --- | --- | --- | --- | --- | --- | --- | --- | --- | --- | --- |
|  |  |  |  |  |  | **Age-adjusted Fully-adjusted** | | | | | |  | **Age-adjusted Fully-adjusted** | | | | | |  | **Age-adjusted Fully-adjusted** | | | | | |  | **Age-adjusted Fully-adjusted** | | | | | |
|  |  | **N** | **Deaths** |  |  | **HR** | **95%** | **CI** | **HR** | **95%** | **CI** |  | **HR** | **95%** | **CI** | **HR** | **95%** | **CI** |  | **HR** | **95%** | **CI** | **HR** | **95%** | **CI** |  | **HR** | **95%** | **CI** | **HR** | **95%** | **CI** |
| **Men** |  |  |  |  |  |  |  |  |  |  |  |  |  |  |  |  |  |  |  |  |  |  |  |  |  |  |  |  |  |  |  |  |
| Past drinking |  |  |  |  |  |  |  |  |  |  |  |  |  |  |  |  |  |  |  |  |  |  |  |  |  |  |  |  |  |  |  |  |
| Stable non-drinker |  | 45 | 6 |  |  | 0.80 | [0.35 | 1.79] | 0.84 | [0.37 | 1.89] |  | 0.74 | [0.24 | 2.32] | 0.77 | [0.25 | 2.43] |  | 1.12 | [0.36 | 3.55] | 1.17 | [0.37 | 3.72] |  | NA |  | NA |  |  |  |
| Stable drinker |  | 2046 | 282 |  |  | 1.00 | . | . | 1.00 | . | . |  | 1.00 | . | . | 1.00 | . | . |  | 1.00 | . | . | 1.00 | . | . |  | 1.00 | . | . | 1.00 | . | . |
| Cut down |  | 1524 | 255 |  |  | 1.09 | [0.92 | 1.29] | 1.04 | [0.87 | 1.24] |  | 1.19 | [0.94 | 1.50] | 1.10 | [0.87 | 1.40] |  | 1.13 | [0.84 | 1.50] | 1.05 | [0.78 | 1.42] |  | 0.98 | [0.67 | 1.43] | 1.03 | [0.70 | 1.51] |
| Ex-drinker |  | 512 | 103 |  |  | 1.47 | [1.17 | 1.84] | 1.25 | [0.99 | 1.57] |  | 1.57 | [1.15 | 2.14] | 1.27 | [0.93 | 1.75] |  | 1.55 | [1.06 | 2.27] | 1.29 | [0.88 | 1.90] |  | 1.21 | [0.72 | 2.01] | 1.10 | [0.66 | 1.85] |
| N |  | 4127 | 646 |  |  | 4127 |  |  | 4127 |  |  |  | 4127 |  |  | 4127 |  |  |  | 4127 |  |  | 4127 |  |  |  | 4127 |  |  | 4127 |  |  |
| Person yrs (000s) |  |  |  |  |  | 24.8 |  |  | 24.8 |  |  |  | 24.8 |  |  | 24.8 |  |  |  | 24.8 |  |  | 24.8 |  |  |  | 24.8 |  |  | 24.8 |  |  |
| N. of deaths |  |  |  |  |  | 646 |  |  | 646 |  |  |  | 348 |  |  | 348 |  |  |  | 227 |  |  | 227 |  |  |  | 131 |  |  | 131 |  |  |
|  |  |  |  |  |  |  |  |  |  |  |  |  |  |  |  |  |  |  |  |  |  |  |  |  |  |  |  |  |  |  |  |  |
| **Women** |  |  |  |  |  |  |  |  |  |  |  |  |  |  |  |  |  |  |  |  |  |  |  |  |  |  |  |  |  |  |  |  |
| Past drinking |  |  |  |  |  |  |  |  |  |  |  |  |  |  |  |  |  |  |  |  |  |  |  |  |  |  |  |  |  |  |  |  |
| Stable non-drinker |  | 424 | 32 |  |  | 1.27 | [0.86 | 1.89] | 1.18 | [0.79 | 1.75] |  | 1.43 | [0.85 | 2.41] | 1.28 | [0.76 | 2.17] |  | 1.21 | [0.58 | 2.55] | 1.04 | [0.49 | 2.20] |  | 0.43 | [0.06 | 3.29] | 0.42 | [0.05 | 3.25] |
| Stable drinker |  | 2818 | 120 |  |  | 1.00 | . | . | 1.00 | . | . |  | 1.00 | . | . | 1.00 | . | . |  | 1.00 | . | . | 1.00 | . | . |  | 1.00 | . | . | 1.00 | . | . |
| Cut down |  | 1301 | 68 |  |  | 1.13 | [0.84 | 1.52] | 1.08 | [0.80 | 1.47] |  | 1.08 | [0.70 | 1.66] | 1.03 | [0.66 | 1.60] |  | 1.16 | [0.65 | 2.04] | 1.12 | [0.62 | 2.00] |  | 2.04 | [0.98 | 4.24] | 1.89 | [0.89 | 3.99] |
| Ex-drinker |  | 468 | 45 |  |  | 1.75 | [1.23 | 2.47] | 1.56 | [1.10 | 2.22] |  | 2.40 | [1.56 | 3.71] | 2.10 | [1.36 | 3.25] |  | 2.49 | [1.40 | 4.43] | 2.17 | [1.21 | 3.90] |  | 1.25 | [0.36 | 4.38] | 1.03 | [0.29 | 3.64] |
| N |  | 5011 | 265 |  |  | 5011 |  |  | 5011 |  |  |  | 5011 |  |  | 5011 |  |  |  | 5011 |  |  | 5011 |  |  |  | 5011 |  |  | 5011 |  |  |
| Person yrs (000s) |  |  |  |  |  | 32.5 |  |  | 32.5 |  |  |  | 32.5 |  |  | 32.5 |  |  |  | 32.5 |  |  | 32.5 |  |  |  | 32.5 |  |  | 32.5 |  |  |
| N. of deaths |  |  |  |  |  | 265 |  |  | 265 |  |  |  | 142 |  |  | 142 |  |  |  | 79 |  |  | 79 |  |  |  | 33 |  |  | 33 |  |  |

Covariates in fully-adjusted model are: age, education, marital status, economic activity, asset score, subjective hardship score, smoking, physical activity, BMI, and depressive symptoms.

Pre-specified alcohol-related causes of death include external causes (ICD-10 codes: F10, S00-T99, V00-Y99), liver disease (B15-19 K70-77, I85), liver cancer (C22), cancer of upper aerodigestive tract (C00-15, 32), tuberculosis(A15-19, B90), pneumonia and other relevant infectious disease (J00-39, J60-98), non-MI acute IHD (I24), non-neoplastic pancreatic disease (K85-86), and relevant ill-specified disease (R00-99) as in Zaridze et al. (14).

# Population attributable risk fraction (PARF) calculations

Population attributable risk fractions (PARF) were calculated using standard formulas with light drinkers (<10 g ethanol/day) as the reference group. We used the HRs from Cox regression results (Table 2 in the main text) and prevalence of two categories of exposure (heavy drinking and non-drinking, shown in Table 1 of the main text), assuming that all excess risk in heavy drinkers is due to alcohol and half of the excess risk in non-drinkers was due to former alcohol consumption). We calculated PARF under five different scenarios assuming that 1) non-response was zero, 2) non-response was 40% but exposure prevalence and RR were the same in non-respondents and respondents, 3) non-response was 40% and RR in non-respondents was double that in respondents, 4) non-response was 40%, prevalence of heavy drinking among non-respondents was 30% and RR in non-respondents was double that in respondents, and 5) non-response was 40%, prevalence of heavy drinking among non-respondents was 30%, and RR in non-respondents was quadruple and in respondents double the baseline value.

#

# Appendix Table 11. Population attributable risk fraction (PARF) calculations for alcohol and all-cause mortality under actual and hypothetical scenarios in men in all centers and Novosibirsk

| **Assumptions** | **Non-respondents** | | | | | |  | **Respondents** | | | | | |  | **PARF (%)** |
| --- | --- | --- | --- | --- | --- | --- | --- | --- | --- | --- | --- | --- | --- | --- | --- |
|  | **Heavy drinkers** | | | **Non-drinkers** | | |  | **Heavy drinkers** | | | **Non-drinkers** | | |  |  |
|  | **Non-**  **response**  **(%)** | **Prevalence (%)** | **RR** | **Non-**  **response**  **(%)** | **Prevalence (%)** | **RR** |  | **Response**  **(%)** | **Prevalence (%)** | **RR** | **Response (%)** | **Prevalence (%)** | **RR** |  |  |
| **All men** |  |  |  |  |  |  |  |  |  |  |  |  |  |  |  |
| 1. Non-respondents ignored | 0 | 2.8 | 1.23 | 0 | 12.4 | 1.36 |  | 100 | 2.8 | 1.23 | 100 | 12.4 | 1.36 |  | **2.8** |
| 1. Same exposure prevalence and RRs in non-respondents and respondents | 40 | 2.8 | 1.23 | 40 | 12.4 | 1.36 |  | 60 | 2.8 | 1.23 | 60 | 12.4 | 1.36 |  | **2.8** |
| 1. Same exposure prevalence but double RRs in non-respondents as respondents | 40 | 2.8 | **1.46** | 40 | 12.4 | **1.72** |  | 60 | 2.8 | 1.23 | 60 | 12.4 | 1.36 |  | **3.9** |
| 1. Higher prevalence of heavy drinkers and double RRs in non-respondents as respondents | 40 | **30** | **1.46** | 40 | 12.4 | **1.72** |  | 60 | 2.8 | 1.23 | 60 | 12.4 | 1.36 |  | **8.3** |
| 1. Higher prevalence of heavy drinkers and quadruple RRs in non-respondents, double RR in respondents | 40 | **30** | **1.92** | 40 | 12.4 | **2.44** |  | 60 | 2.8 | **1.46** | 60 | 12.4 | **1.72** |  | **15.3** |
|  |  |  |  |  |  |  |  |  |  |  |  |  |  |  |  |
| **Novosibirsk men** |  |  |  |  |  |  |  |  |  |  |  |  |  |  |  |
| 1. Non-respondents ignored | 0 | 2.3 | 1.60 | 0 | 13.5 | 1.14 |  | 100 | 2.3 | 1.60 | 100 | 13.5 | 1.14 |  | **2.3** |
| 1. Same exposure prevalence and RRs in non-respondents and respondents | 33 | 2.3 | 1.60 | 33 | 13.5 | 1.14 |  | 67 | 2.3 | 1.60 | 67 | 13.5 | 1.14 |  | **2.3** |
| 1. Same exposure prevalence but double RRs in non-respondents as respondents | 33 | 2.3 | **2.20** | 33 | 13.5 | **1.28** |  | 67 | 2.3 | 1.60 | 67 | 13.5 | 1.28 |  | **3.0** |
| 1. Higher prevalence of heavy drinkers and double RRs in non-respondents as respondents | 33 | **30** | **2.20** | 33 | 13.5 | **1.28** |  | 67 | 2.3 | 1.60 | 67 | 13.5 | 1.28 |  | **12.3** |
| 1. Higher prevalence of heavy drinkers and quadruple RRs in non-respondents, double RR in respondents | 33 | **30** | **3.40** | 33 | 13.5 | **1.56** |  | 67 | 2.3 | **2.20** | 67 | 13.5 | **1.28** |  | **21.8** |

Light drinkers (<10 g ethanol/day) were used as reference group for PARF calculations.

Figures in bold are estimates of exposure prevalence or relative risk under hypothetical scenarios.
